# Electrochemical Bromination of Arenes in a 200% Cell

**DOI:** 10.1021/acs.joc.4c01086

**Published:** 2024-09-25

**Authors:** Sara Torabi, Mahdi Jamshidi, Gerhard Hilt

**Affiliations:** Institute of Chemistry, Carl von Ossietzky University Oldenburg, Carl-von-Ossietzky-Str. 9-11, 26129 Oldenburg, Germany

## Abstract

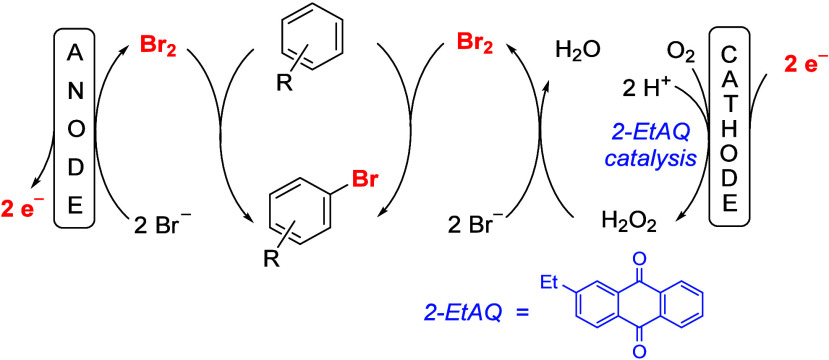

Herein, we describe the investigation of electrochemical bromination of electron-rich arenes in a 200% cell. For this application, at first, the influence of an excess of supporting electrolyte (Bu_4_NBr) on chemical bromination was investigated. The application of >4.0 equiv of Bu_4_NBr proved to enhance the regioselectivity of the bromination process for O- and N-substituted arenes considerably. The linear paired electrolysis was then optimized upon these insights, and a number of electron-rich arenes could be brominated in high yields with excellent regioselectivity. The use of O_2_ as a sacrificial starting material in combination with 2-ethylanthraquinone as a catalyst leads to the enhanced formation of H_2_O_2_ at the cathode, resulting in current efficiencies >150% for a considerable number of examples.

## Introduction

**Scheme 1:**
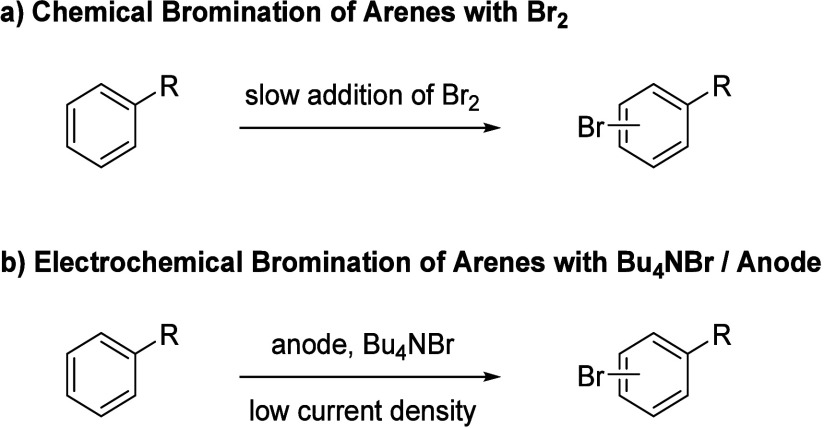
Chemical Bromination (a) and Electrochemical Bromination (b) of Electron-Rich Arenes

The bromination of electron-rich arenes with elemental Br_2_ is a standard procedure in organic synthesis for the functionalization of aromatic compounds and a fundamental reaction discussed in every organic chemical textbook (Scheme 1a). Other brominating agents have also been reported, which have in common that they are a source of Br^**+**^ for the electrophilic aromatic substitution reaction.^1^ However, the needed Br_2_ is commercially readily available but hazardous and unpleasant to work with. On the other hand, Br_2_ can also be easily prepared by anodic oxidation of bromide anions when a suitable and much less harmful supporting electrolyte, e.g., NaBr or Bu_4_NBr, is utilized (Scheme 1b).^2^ Accordingly, there have been several reports, where electron-rich arenes were converted under electrochemical conditions to afford the desired brominated products.^3^ Some recent contributions should be mentioned here, such as the catalyst-free electrochemical anodic bromination of electron-rich (hetero)arenes at 80 °C in an undivided cell, utilizing NaBr/HBr as the supporting electrolyte, as reported by Lei and co-workers.^4^ Xiang and co-workers accomplished the electrochemical bromination of electron-rich aromatic rings and pyridine derivatives using Bu_4_NBr as the source for Br_2_ in dichloromethane as the solvent in an undivided cell at room temperature.^5^ Also, a two-phase electrolysis for the bromination of electron-rich aromatic rings at 0 °C was disclosed by Raju et al.^6^ The aqueous phase contained 50–60% NaBr and 5% HBr, while the organic starting materials were dissolved in the organic chloroform phase. A microflow electrochemical cell was described by Liu and Rivera^7^ for the electrochemical bromination of drug molecules and advanced intermediates, such as *cytidine*, *uridine*, and *tenofovir*, in aqueous NaBr solution.

For the bromination of arenes, transition metal catalysts have been the subject of recent reports. In this respect, Fang, Huang, and Mei reported the electrochemical bromination of quinoline derivatives at the C5 position, which was achieved utilizing NH_4_Br as the supporting electrolyte and Cu(OAc)_2_ as the catalyst in DMF.^8^ Also, Ackermann and co-workers disclosed the electrochemical *meta*-selective bromination of arenes bearing heteroaromatic substituents under electrolyte-free conditions with RuCl_3_·3H_2_O as the catalyst and aqueous HBr in an undivided cell.^9^

The concept of paired electrolysis was also applied to the bromination of aromatic compounds. Under these conditions, the bromide anions are liberated at the cathode upon reductive cleavage of carbon–bromine bonds, and the anodic electrochemical reaction generates the desired Br_2_. In this respect, Lei and co-workers used CHBr_3_, CH_2_Br_2_, and CCl_3_Br as bromine sources in such a paired electrochemical C–H halogenation of imidazopyridine derivatives at elevated temperatures.^10^ Another innovative way for the formation of bromide anions at the cathode was reported by Wei, Hou, and Wang, utilizing a paired electrochemical process from 2-bromoethan-1-ol.^11^ At the cathode, bromide anions were formed, again upon unproductive electrochemical H_2_ formation, releasing ethylene oxide as a neutral side product, and the anodic bromide oxidation then initiated the bromination of (hetero)-arenes.

Herein, we describe our efforts in utilizing a linear paired electrolysis for the bromination of electron-rich arenes.

## Results and Discussion

**Scheme 2:**
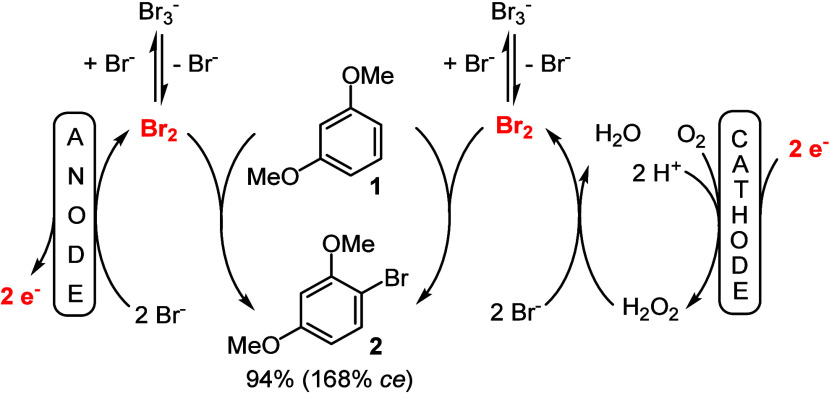
Linear Paired Electrolysis for the Bromination of **1** to **2**

The concept of linear paired electrolysis is that both electrochemical transformations, anodic oxidation, and cathodic reduction, should generate the same product. This paradox can be realized when a sacrificial starting material is used. In principle, the anodic oxidation and cathodic reduction can reach 100% current efficiency so that the current passed through the solution is used twice for the synthesis of the desired product. In other words, if the stoichiometric oxidation of the starting material is a two-electron process, in regular electrolysis, a current of 2.0 *F* is needed. In a linear paired electrolysis with a highly efficient oxidation process and a perfect transformation of the sacrificial starting material and its follow-up reaction, only 1.0 *F* would be needed to reach 100% chemical yield and 200% current efficiency. Thereby, the overall current efficiencies of up to 200% could be reached. There are two different scenarios:

a) An *overall reduction* would be realized when the regular cathodic reduction of the starting material is combined with an anodic oxidation of a sacrificial starting material, thereby producing a stronger reducing agent as the sacrificial starting material itself. Unfortunately, we are not aware of any example where this concept has been realized thus far.
b) An *overall oxidation* is realized when the regular anodic oxidation of the starting material is combined with a cathodic reduction of a sacrificial starting material, generating a stronger oxidizing agent. An example and the outline of such a reaction is shown in Scheme 2.

In 2021, we applied a linear paired electrolysis for the bromination of alkenes, realizing excellent yields and current efficiencies based on the fast follow-up reaction of Br_2_ with alkenes.^12^ In a single example, we also applied a very electron-rich arene (1,3-dimethoxybenzene, **1**) in such a linear paired electrolysis process (Scheme 2) and achieved a very good yield and a high current efficiency (ce) for the desired product **2**, utilizing molecular oxygen as a sacrificial starting material for the formation of H_2_O_2_ at the cathode.^13,14^

A comparison of the chemical and electrochemical bromination reactions outlined in Scheme 1 reveals a significant difference between these two reactions, which is already incorporated in Scheme 2; the bromide concentration is an important parameter in these reactions. Therefore, we provide a short analysis of the two procedures.

### Chemical Bromination

When the bromination of an arene is started by the addition of the first portion of Br_2_ in an appropriate solvent (*t* = 0), the concentration of bromide ions in the solution is zero. Over the course of the electrophilic aromatic substitution, the concentration of bromide ions (or HBr) in the solution steadily increases.

When a tribromide salt, such as pyridinium tribromide (pyH·Br_3_) is used, the concentration of bromide anions is relatively low, according to the equilibrium between (Br^–^ + Br_2_ ⇄ Br_3_^–^) in the solvent applied. However, upon the electrophilic aromatic substitution, the bromide concentration steadily increases twice the amount compared to the bromination with Br_2_.

### Electrochemical Bromination

When the electrolysis is started, the concentration of bromide ions (e.g., from the supporting electrolyte, such as Bu_4_NBr) is relatively high, at least 1.0 equiv with respect to the arene to be functionalized. At the end of the electrolysis, the bromide ion concentration is relatively low when only 1.0 equiv of Bu_4_NBr is used and the starting material is mostly brominated. Also, in the electrochemical version, the relatively high bromide concentration and the low Br_2_ concentration (at low currents) lead to the formation of Br_3_^–^ ions in equilibrium, as outlined before (see also Scheme 2), and represent a conceptional alternative brominating agent compared to the transformations with Br_2_ or with pyH·Br_3_. In the chemical bromination reactions, the bromide ion concentration steadily increases, while in the electrochemical bromination, the bromide concentration decreases.

**Scheme 3:**
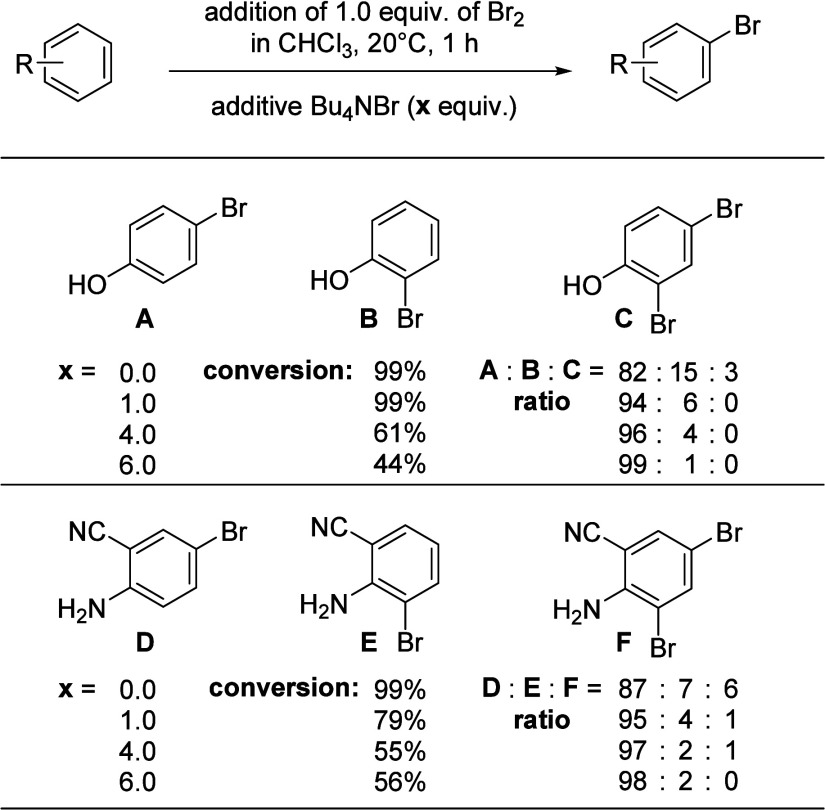
Results of the Chemical Bromination in the Presence of Different Concentrations of Bu_4_NBr

Until now, we found only very few indications in the literature that the bromide concentration has an impact on the bromination of an electron-rich arene under electrochemical conditions.^15^ Therefore, we conducted experiments with selected electron-rich arenes to investigate if this parameter is of relevance in a chemical bromination reaction, mimicking the presence of different supporting electrolyte concentrations (= *x* equiv of Bu_4_NBr). The results for the bromination of the monosubstituted starting material phenol (R = OH) and the disubstituted arene 2-aminobenzonitrile are summarized in Scheme 3.

The chemical bromination of phenol in the absence of Bu_4_NBr led to three brominated products (**A**–**C**) that could be identified by gas chromatography–mass spectrometry (GC-MS) and quantified by GC analysis. With increasing amounts of Bu_4_NBr present in the solution, the selectivity toward the formation of 4-bromophenol **A** steadily increased, which is almost perfect in the presence of 6.0 equiv of Bu_4_NBr, resulting in a ratio of **A**:**B** = 99:1 and the double brominated product **C** was not detected anymore. The excellent regioselectivity for the almost exclusive formation of **A** was associated with a slower conversion of phenol with increasing amounts of Bu_4_NBr and led only to 44% conversion after 1 h of reaction time. Nevertheless, these are the results after 1 h reaction time, and therefore, we assume that the equilibrium between Br^–^ + Br_2_ ⇄ Br_3_^–^ reduces the reactivity toward the substrate but increases the selectivity toward the formation of product **A** in high excess. Also, the tribromide anion represents a different brominating agent with altered bond lengths and an electrophilic site at the ends of the molecule, and therefore, altered regioselectivities can be observed.^16^ In line with the results for phenol are those for 2-aminobenzonitrile, which led to the formation of the brominated products **D**–**F**. For this substrate, the formation of product **D** is highly favored upon the cooperative directing group effects of the NH_2_ and the CN group, but still, the reactivity is reduced, whereas the selectivity toward the formation of **D** is again enhanced with increasing amounts of Bu_4_NBr present in solution. These results mimic the “expected” outcome of an electrochemical bromination with respect to the regioselectivity of electron-rich arenes when an excess of supporting electrolyte Bu_4_NBr is present, and the electrolysis is conducted at low to moderate current densities to generate Br_2_ relatively slow. As a compromise, we decided to use an excess of the supporting electrolyte (4.0 equiv of Bu_4_NBr) when the parameters for a linear paired electrolysis for the bromination of electron-rich arenes were optimized.

**Scheme 4:**
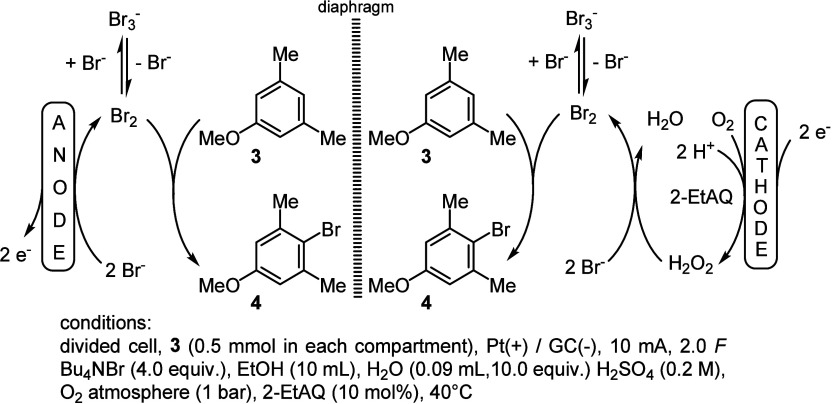
Outline of the Linear Paired Electrolysis for the Bromination of **3** toward **4** in a Divided Cell for the Determination of the Efficiency in the Cathode and Anode Compartment

One can easily anticipate that the optimization for the formation of the desired brominated product **4** (see Scheme 4) at the cathode will be the decisive factor in reaching current efficiencies >100%. Therefore, the setup of a divided electrochemical cell was chosen to determine the efficiency of the reaction in the cathode as well as in the anode compartment, independently. In fact, the bromination of 3 in the anode compartment gave the desired product 4 in >95% yield (by GC) in almost all reactions. The setup for optimization of the cathode process is shown in Scheme 4.

The results for the optimization of the solvent, electrode material, bromide source, and additives are summarized in Table 1.

**Table 1:** Results of Optimization of the Bromination of Arene **3** in the Cathode Compartment^a^

no.	variation of conditions	cathodic yield^b^
1	none	71%
2	EtOH @ 0 °C	31%
3	EtOH @ 25 °C	40%
4	EtOH @ 40 °C	49%
5	EtOH @ 50 °C	39%
6	CH_3_CN @ 0 °C	27%
7	CH_3_CN @ 25 °C	36%
8	DMF @ 25 °C	3%
9	Au cathode	4%
10	Pt cathode	23%
11	Pd cathode	17%
12	BDD cathode	14%
13	NaBr	9%
14	NH_4_Br	10%
15	EtOH @ 40 °C + 2-EtAQ (20%)^c^	56%
16	EtOH @ 50 °C + 2-EtAQ (10%)^c^	67%
17	EtOH @ 60 °C + 2-EtAQ (10%)^c^	56%

a The reaction conditions are specified in Scheme 4. The reaction mixture was saturated with O_2_ for 5 min at 40 °C prior to electrolysis.

b The yield was determined by GC analysis of the crude reaction mixture.

c 2-EtAQ = 2-ethylanthraquinone (see Scheme 5).

The survey to find a suitable solvent revealed that ethanol is superior to acetonitrile and DMF and that the temperature of 40 °C gave the best results (entries 2–8).^17^ As the cathode material, glassy carbon is superior to gold, platinum, palladium, or a boron-doped diamond electrode (BDD) (entries 9–12). Also, Bu_4_NBr gave the best result concerning the supporting electrolyte and the bromide source, while NaBr and NH_4_Br proved to be less effective, resulting in only low yields (entries 13/14) caused by their low solubility and low conductivity in ethanol.^18^ The efficiency of the H_2_O_2_ production in the cathode compartment was further enhanced when 2-ethylanthraquinone (2-EtAQ)^19^ was added in the presence of H_2_SO_4_ (entries 15–17), while higher temperatures at 50/60 °C or higher catalyst loading did not improve the yield of **4**.

With the optimized reaction conditions in hand, we investigated the linear paired electrolysis of selected electron-rich arenes in an undivided cell under an O_2_ atmosphere, utilizing Bu_4_NBr (4.0 equiv) as a supporting electrolyte in the presence of 2-EtAQ (10 mol %) as a catalyst in acidified ethanol solution at 40 °C. In this investigation, we focused our attention on the bromination of phenols, anisole, and aniline derivatives. The results for the linear paired electrolysis of electron-rich arenes are summarized in Scheme 5.

**Scheme 5:**
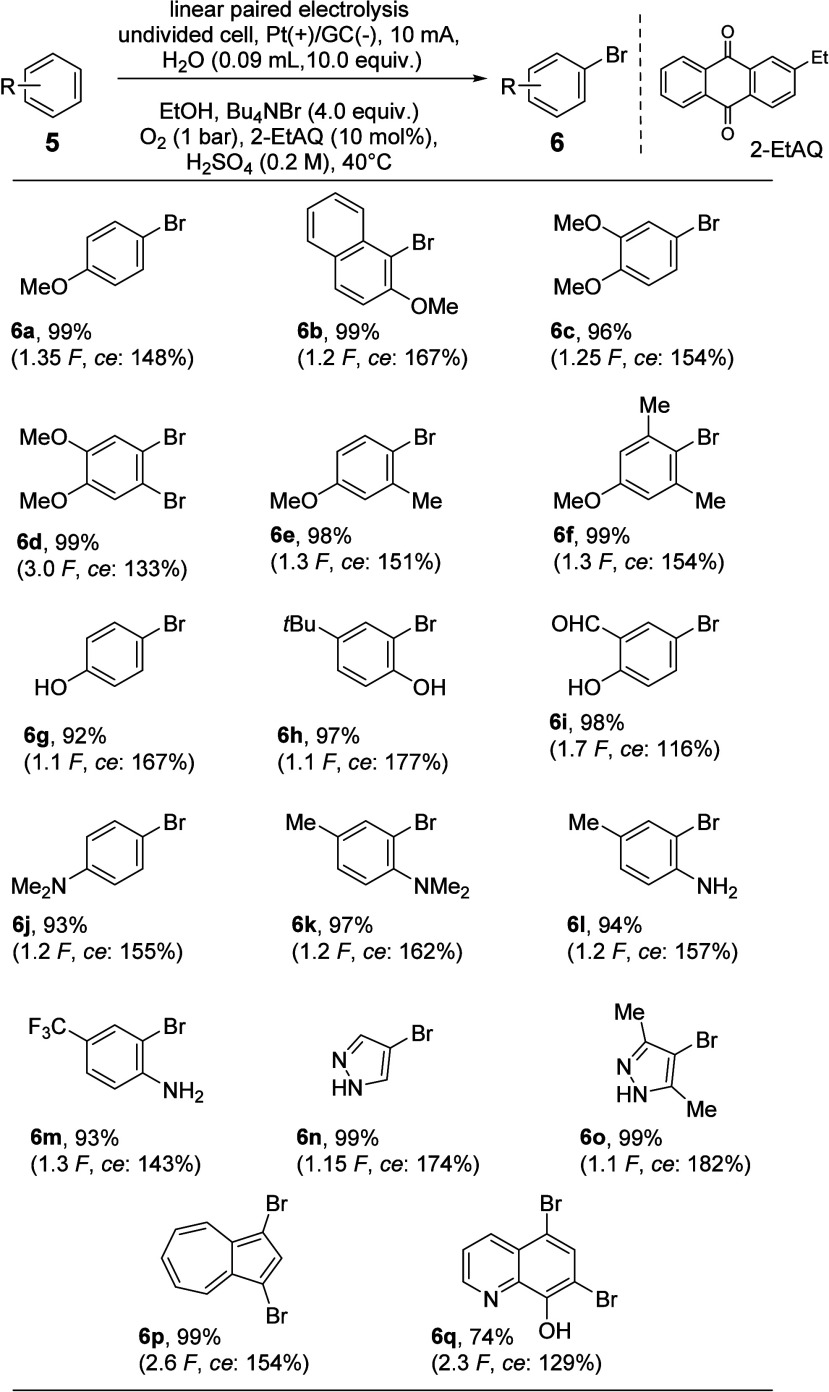
Results of the Linear Paired Electrolysis for the Bromination of Electron-Rich Arenes of Type **5**

The theoretical current needed for the transformation (1.0 *F*) had to be adjusted for each substrate individually to compensate for the cathodic process, which exhibited a lower efficiency than 100%. However, anisole and related compounds could be brominated in the linear paired electrolysis with high chemical yields >96% (**6a**–**6f**). The regioselectivities are exclusive for many examples, showing only traces of other regioisomers by GC or GC-MS analysis when unsymmetrical starting materials were applied. The current efficiencies (ce) are also high, exceeding 150% ce for many examples, such as for **6b** reaching 167% ce. A double bromination could be realized for the formation of **6d**, and the product could be obtained with good overall chemical yield (99%) and current efficiency (133% ce). When phenol derivatives were applied (**6g**–**6i**), similar results were obtained with yields of >92%, exclusive regioselectivities, and current efficiencies up to 177% ce. The situation is similar concerning the parameters in focus in this investigation for aniline and *N*,*N*-dimethyl aniline derivatives (**6j**–**6m**). Noteworthy is the substrate **6m** bearing an electron-deficient substituent (CF_3_), which was brominated with 143% ce, but in a good yield of 93%, utilizing just a small excess (1.3 *F*) of the theoretical current needed. The heterocycle 1*H*-pyrazole and the corresponding 3,5-dimethyl derivative led to the formation of **6n** and **6o**, using only a small excess of current (1.10–1.15 *F*), and were isolated in almost quantitative yield.^19^ Based on our interest in azulene chemistry, we also applied azulene in the linear paired electrolysis to generate the dibrominated product **6p**. The bromination occurred selectively on the electron-rich five-membered ring in the 1- and 3-position, and the process needed only 2.6 *F*, resulting in 154% ce to generate the desired product **6p** in 99% yield. Last but not least, quinolin-8-ol was subjected to a double bromination in a linear paired electrolysis, and the desired product **6q** was generated in an acceptable yield of 74% and current efficiency of 129%; as expected, the bromination chemoselectively took place on the more electron-rich phenol part of the starting material.^20^

Caution: Aryl bromides are potentially hazardous and can cause skin irritation; avoid direct contact.

While these results are very promising, there is a serious limitation on the horizon. For the bromination of electron-deficient arenes, a *Lewis*-acid activation would be needed. The combination of a suitable *Lewis* acid with bromide anions from the supporting electrolyte in large excess is incompatible and resembles a serious limiting factor.

## Conclusions

In summary, we have accomplished an effective electrochemical bromination of electron-rich (hetero)arenes in a linear paired electrolysis in high yields and high current efficiencies (often) >150% ce. The use of 2-EtAQ as a catalyst for the generation of hydrogen peroxide at the cathode proved to be beneficial under acidic conditions. In the presence of an excess of supporting electrolyte Bu_4_NBr (4.0 equiv) in the chemical as well as in the electrochemical bromination, excellent regioselectivities were observed, and the selectivity for the bromination in the para-position of aniline and phenol derivatives was significantly enhanced.

## Data Availability

The data underlying this study are available in the published article and its Supporting Information.

## Supporting Information Available

General information, optimization of electrolysis, experimental details, spectroscopic data, NMR spectra (PDF)
