# Supplementary material for: Electrochemical Bromination of Arenes in a 200% Cell
Source: J Org Chem. 2024 Sep 25;89(19):13953–8. doi: 10.1021/acs.joc.4c01086 (PMC11460727; doi:10.1021/acs.joc.4c01086)
Supplement: Supplementary file 1 — jo4c01086_si_001.pdf [file jo4c01086_si_001.pdf]

Supporting Information

The Electrochemical Bromination of Arenes in a 200% Cell

Sara Torabi, Mahdi Jamshidi and Gerhard Hilt*

Institute of Chemistry, Carl von Ossietzky University Oldenburg, Carl-von-Ossietzky-Str. 9-11, 26129 Oldenburg, Germany

Email: Gerhard.Hilt@uni-oldenburg.de

Supporting Information

Table of content

1. General Information S2

2. Electrosynthesis S3

2.1. Optimization of the reaction conditions on cathodic part S3

2.2 General procedure for the optimization of the cathodic part S4

2.3. General procedure (GPA) for the electrochemical bromination S4

3. Analytical section S5

3.1. Analytical data of synthesized bromoarenes S5

3.2. NMR spectra of all synthesized compounds S12

4. References S29

1. General Information

All solvents were commercially available and have been distilled under reduced pressure prior to use. Solvents were dried over 3 Å molecular sieves. All chemicals or reagents were purchased from commercial suppliers and used without further purification, if not otherwise stated, or were prepared according to known literature procedures. If water or air sensitive compounds have been used, the experiments were carried out in heat gun dried glassware using conventional SCHLENK techniques under nitrogen atmosphere. Electrochemical reactions were carried out using an AIM-TTI Instruments MX100T power supply. These reactions were performed in an undivided and divided cell (**Figure S1**), equipped with a stirring bar, a platinum anode (1.60 · 3.40 cm^2, active surface: 3.20 cm^2) and a glassy carbon cathode (1.50 · 3.50 cm^2, active surface: 3.0 cm^2) (distance between the electrodes: 0.9 cm). All known compounds were characterized by ^{1}H and ^{13}C NMR. All unknown compounds were identified by ^{1}H NMR, ^{13}C NMR, and IR.

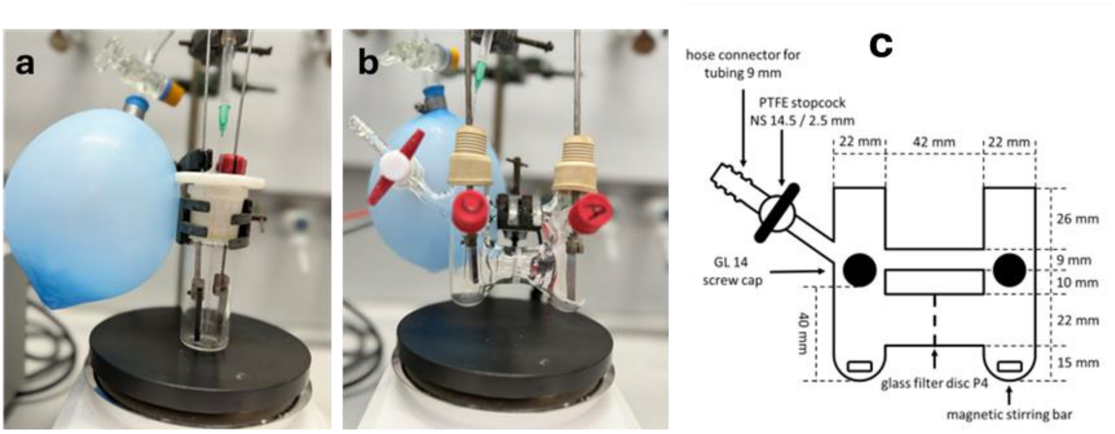

Figure S1: (a) Undivided and (b) divided electrolysis cell, equipped with a platinum anode and a glassy carbon cathode. (c) Drawing of the divided electrolysis cell.

NMR spectroscopy: NMR spectra were recorded either on a Bruker Avance 300 (300 MHz), on a Bruker Avance III (500 MHz) or on a Bruker Avance DRX (500 MHz). Chemical shifts are reported in parts per million (ppm). The spectra are referenced to the residual solvent peak of $CDCl_3$. In the ^{1}H NMR spectra this corresponds with the singlet of the solvent signal of $CDCl_3$ at δ = 7.26 ppm. The ^{13}C NMR spectra were referenced to the central line of the triplet of $CDCl_3$ at δ = 77.16 ppm.

IR Spectroscopy: The IR spectra were obtained with a Shimadzu IR Spirit with a QATR-S cell. The wave numbers λ^{-1} are quoted in reciprocal centimeters (cm^{-1}).

2. Electrosynthesis

2.1. Optimization of different parameters on cathodic bromination of aromatic rings

2.2 General procedure for the optimization of the cathodic part

All experiments were conducted in a divided cell. Anodic bromination was carried out at the surface of a Pt electrode using a solution containing tetrabutylammonium bromide (TBAB) (2.00 mmol, 4.0 equiv.) and anhydrous solvent (10 mL). The cathodic bromination was optimized based on reported experiments (Tables 1-4).

Table S1. Optimization of Solvent and Temperature.

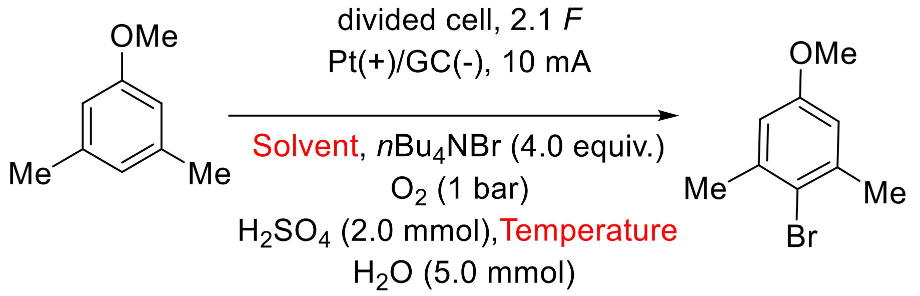

Entry	Solvents and Temperatures	Yield (Cathode)[a]
1	EtOH at 0 °C	31%
2	CH_3CN at 0 °C	27%
3	EtOH at 25 °C	40%
4	CH_3CN at 25 °C	36%
5	DMF at 25 °C	3%
6	EtOH at 40 °C	49%

[a] The yield was determined by GC analysis.

Table S2. Optimization of Bromide Source.

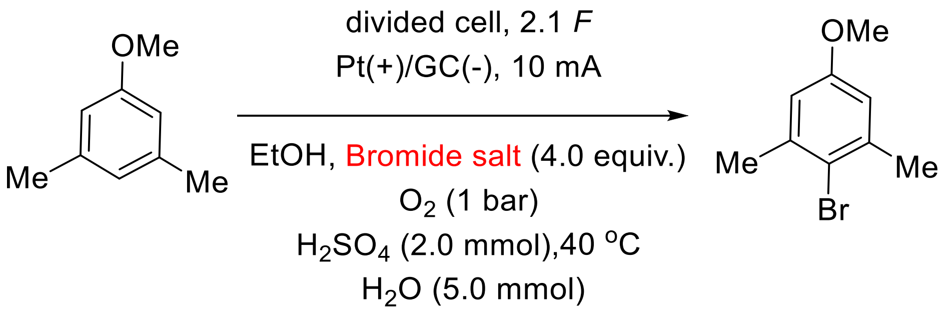

Entry	Bromide Source	Yield (Cathode)
1	NaBr as Br^- source	9%
2	NH_4Br as Br^- source	10%

Table S3. Optimization of Cathode Material.

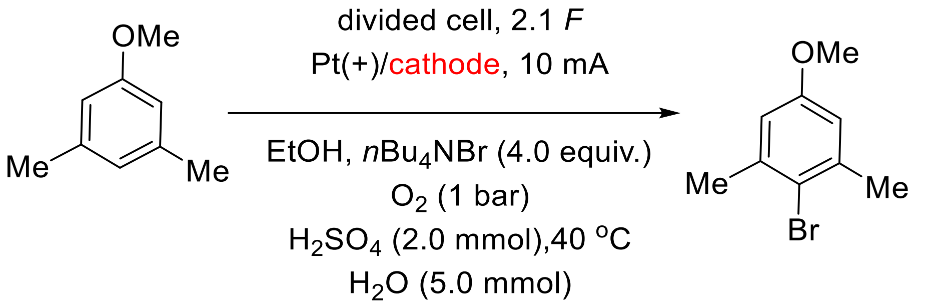

Entry	Cathode	Yield (Cathode)
1	Au as cathode electrode	4%
2	Al as cathode electrode	3%
3	Pt as cathode electrode	23%
4	Pd as cathode electrode	17%
5	BDD as cathode electrode	14%

Table S4. Optimization of Catalyst.

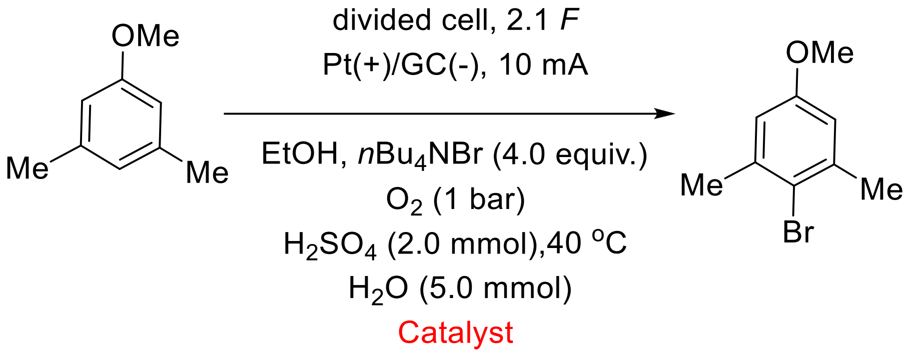

Entry	Catalyst	Yield (Cathode)
1	10 mol % 2-EtAQ [b]	71%
2	20 mol % 2-EtAQ [b]	56%
3	10 mol % 2-EtAQ at 50 °C	67%
4	10 mol % 2-EtAQ at 60 °C	56%

[b] 2-EtAQ = 2-ethylanthraquinone

2.3. General procedure (GPA) for the electrochemical bromination

First, the tetrabutylammonium bromide (TBAB) (2.00 mmol, 4.0 equiv.) as bromine source and 2-ethylanthracene-9,10-dione (10 mol%) were weighed into an undivided cell and dissolved in anhydrous ethanol (10 mL). Afterwards, water (0.09 mL, 90 mg, 5.0 mmol, 10 equiv.), conc. sulfuric acid (0.11 mL, 196 mg, 2.00 mmol) and the corresponding arene (0.50 mmol, 1.0 equiv.) were added and the reaction mixture was heated to 40 °C (oil bath). At this temperature, the solution was saturated with oxygen by

bubbling the gas through the solution for 5 minutes. Afterwards, the reaction mixture was electrolyzed under constant current (10 mA, Pt anode, glassy carbon cathode) at 40 °C and with an oxygen atmosphere. The reaction mixture was diluted with saturated aqueous $Na_2S_2O_3$ solution (30 mL) and extracted with n-pentane (3 × 20 mL). The combined organic layers were dried ($MgSO_4$) and filtered. The residue was either submitted to column chromatography (SiO_2) or all volatile compounds were removed under reduced pressure to furnish the respective brominated arenes.

Caution: Aryl bromides are potentially hazardous, toxic and irritating; avoid skin contact or inhalation.

3. Analytical section

3.1. Analytical data of synthesized bromoarenes

1-Bromo-4-methoxybenzene (6a)

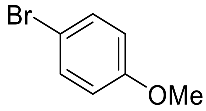

According to the General Procedure, anisole (0.50 mmol, 1.0 equiv.) and tetrabutylammonium bromide (645 mg, 2.00 mmol, 4.0 equiv.) were converted to furnish product **6a** as a colorless liquid (92 mg, 0.495 mmol, 99%, 148% CE) after 1.35 *F* (108 min, 10 mA).

^{1}H NMR (500 MHz, $CDCl_3$): δ = 3.70 (s, 3H), 6.63 (d, *J* = 9.0 Hz, 2H), 7.22 (d, *J* = 9.0 Hz, 2H) ppm. **^{13}C{1H} NMR** (125 MHz, $CDCl_3$): δ = 158.9, 132.3, 115.8, 112.8, 55.4 ppm. **GC/MS**: 188 (99), 186 (100), 171 (45), 143 (43), 92 (21), 63 (68).

The spectroscopic values are in accordance with literature values.[6]

1-Bromo-2-methoxynaphthalene (6b)

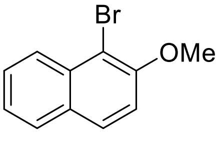

According to the General Procedure, 2-methoxynaphthalene (0.50 mmol, 1.0 equiv.) and tetrabutylammonium bromide (645 mg, 2.00 mmol, 4.0 equiv.) were converted to furnish product **6b** as a white solid (117 mg, 0.495 mmol, 99%, 167% CE) after 1.2 *F* (96 min, 10 mA).

^{1}H NMR (500 MHz, $CDCl_3$): δ = 3.95 (s, 3H), 7.19 (d, *J* = 9.0 Hz, 1H), 7.31 (ddd, *J* = 8.0, 6.8, 1.2 Hz, 1H), 7.48 (ddd, *J* = 8.4, 6.8, 1.3 Hz, 1H), 7.72 (dd, *J* = 16.3, 8.3 Hz, 2H), 8.15 (d, *J* = 8.6 Hz, 1H) ppm. **^{13}C{1H} NMR** (125 MHz, $CDCl_3$): δ = 153.8, 133.2, 129.9, 129.0, 128.0, 127.7, 126.2, 124.3, 113.7, 108.8, 57.1 ppm. **GC/MS**: 238 (98), 236 (100), 193 (81), 142 (9), 127(37), 114 (77), 88(24).

The spectroscopic values are in accordance with literature values.[8]

4-Bromo-1,2-dimethoxybenzene (6c)

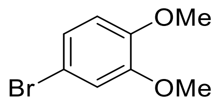

According to the General Procedure, 1,2-dimethoxybenzene (0.50 mmol, 1.0 equiv.) and tetrabutylammonium bromide (645 mg, 2.00 mmol, 4.0 equiv.) were converted to furnish product **6c** as a colorless oil (104 mg, 0.48 mmol, 96%, 154% CE) after 1.25 *F* (100 min, 10 mA).

^{1}H NMR (500 MHz, $CDCl_3$): δ = 3.76 (s, 3H), 3.77 (s, 3H), 6.65 (d, *J* = 8.54 Hz, 1H), 6.90 (d, *J* = 2.35 Hz, 1H), 6.94 (dd, *J* = 8.52, 2.27 Hz, 1H) ppm. **^{13}C{1H} NMR** (125 MHz, $CDCl_3$): δ = 149.8, 148.4, 123.4, 114.9, 112.7, 112.6, 56.1, 56.0 ppm. **GC/MS**: 218 (63), 216 (64), 201 (30), 173 (13), 94 (100), 79 (47).

The spectroscopic values are in accordance with literature values.[2]

1,2-Dibromo-4,5-dimethoxybenzene (**6d**)

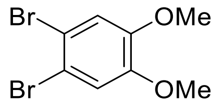

According to the General Procedure, 1,2-dimethoxybenzene (0.50 mmol, 1.0 equiv.) and tetrabutylammonium bromide (645 mg, 2.00 mmol, 4.0 equiv.) were converted to furnish product **6d** as a dark yellow oil (146 mg, 0.495 mmol, 99%, 133% CE) after 3.0 *F* (241 min, 10 mA).

^{1}H NMR (500 MHz, $CDCl_3$): δ = 3.77 (s, 6H), 6.98 (s, 2H) ppm. **^{13}C{1H} NMR** (125 MHz, $CDCl_3$): δ = 149.0, 116.1, 114.8, 56.3 ppm. **GC/MS**: 296 (100), 294 (51), 281 (45), 253 (17), 172 (57), 157 (26), 143 (17), 131 (13), 93 (25).

The spectroscopic values are in accordance with literature values.[12]

1-Bromo-4-methoxy-2-methylbenzene (6e)

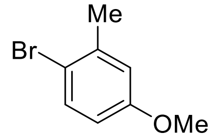

According to the General Procedure, 1-methoxy-3-methylbenzene (0.50 mmol, 1.0 equiv.) and tetrabutylammonium bromide (645 mg, 2.00 mmol, 4.0 equiv.) were converted to furnish product **6e** as a colorless liquid (98 mg, 0.49 mmol, 98%, 151% CE) after 1.3 *F* (104 min, 10 mA).

^{1}H NMR (500 MHz, $CDCl_3$): δ = 2.27 (s, 3H), 3.67 (s, 3H), 6.52 (dd, *J* = 8.8, 3.1 Hz, 1H), 6.69 (d, *J* = 2.9 Hz, 1H), 7.30 (d, *J* = 8.7 Hz, 1H) ppm. **^{13}C{1H} NMR** (125 MHz, $CDCl_3$): δ = 158.9, 138.8, 132.8, 116.5, 115.4, 113.0, 55.4, 23.1 ppm. **GC/MS**: 202 (100), 200 (100), 185 (27), 157 (28), 121 (50), 91 (61), 78(85).

The spectroscopic values are in accordance with literature values.[7]

2-Bromo-5-methoxy-1,3-dimethylbenzene (6f)

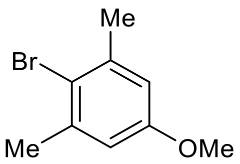

According to the General Procedure, 1-methoxy-3,5-dimethylbenzene (0.50 mmol, 1.0 equiv.) and tetrabutylammonium bromide (645 mg, 2.00 mmol, 4.0 equiv.) were converted to furnish product **6f** as a colorless liquid (106 mg, 0.495 mmol, 99%, 154% CE) after 1.3 *F* (104 min, 10 mA).

^{1}H NMR (500 MHz, $CDCl_3$): δ = 2.29 (s, 6H), 3.66 (s, 3H), 6.55 (s, 2H) ppm. **^{13}C{1H} NMR** (125 MHz, $CDCl_3$): δ = 158.1, 139.1, 118.2, 113.9, 55.3, 24.0 ppm. **GC/MS**: 216 (100), 214 (100), 199 (13), 171 (29), 135 (51), 91 (84).

The spectroscopic values are in accordance with literature values.[1]

4-Bromophenol (6g)

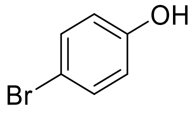

According to the General Procedure, phenol (0.50 mmol, 1.0 equiv.) and tetrabutylammonium bromide (645 mg, 2.00 mmol, 4.0 equiv.) were converted to furnish product **6g** as a white solid (79 mg, 0.46 mmol, 92%, 167% CE) after 1.1 *F* (88 min, 10 mA).

^{1}H NMR (500 MHz, $CDCl_3$): δ = 5.55 (s, 1H), 6.59 (d, *J* = 8.9 Hz, 2H), 7.20 (d, *J* = 9.0 Hz, 2H) ppm. **^{13}C{1H} NMR** (125 MHz, $CDCl_3$): δ = 154.3, 132.6, 117.3, 113.3 ppm. **IR** (ATR): λ^{-1} = 3335, 1590, 1487, 1432, 1334, 1240, 1217, 1172, 1115, 1068, 1004, 934, 824, 808, 602, 502. **GC/MS**: 174 (84), 172(88), 143 (4), 93 (42), 65 (100), 50 (13).

The spectroscopic values are in accordance with literature values.[9]

2-Bromo-4-(*tert.*-butyl)phenol (6h)

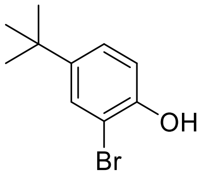

According to the General Procedure, 4-(*tert.*-butyl)phenol (0.50 mmol, 1.0 equiv.) and tetrabutylammonium bromide (645 mg, 2.00 mmol, 4.0 equiv.) were converted to furnish product **6h** as a dark yellow oil (110 mg, 0.485 mmol, 97%, 177% CE) after 1.1 *F* (88 min, 10 mA).

^{1}H NMR (500 MHz, $CDCl_3$): δ = 7.36 (d, *J* = 2.2 Hz, 1H), 7.15 (dd, *J* = 8.5, 2.4 Hz, 1H), 6.87 (d, *J* = 8.6 Hz, 1H), 5.27 (s, 1H), 1.20 (s, 9H) ppm. **^{13}C{1H} NMR** (125 MHz, $CDCl_3$): δ = 149.9, 145.1, 128.8, 126.2, 115.6, 109.9, 34.2, 31.4 ppm. **GC/MS**: 230 (26), 228 (27), 213 (100), 187 (13), 134 (77), 91 (9), 77 (15).

The spectroscopic values are in accordance with literature values.[13]

5-Bromo-2-hydroxybenzaldehyde (6i)

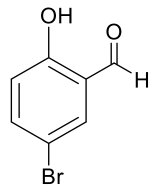

According to the General Procedure, 1-(2-hydroxyphenyl)ethan-1-one (0.50 mmol, 1.0 equiv.) and tetrabutylammonium bromide (645 mg, 2.00 mmol, 4.0 equiv.) were converted to furnish product **6i** as a white solid (98 mg, 0.49 mmol, 98%, 116% CE) after 1.7 *F* (137 min, 10 mA).

^{1}H NMR (500 MHz, $CDCl_3$): δ = 6.92 (d, *J* = 8.9 Hz, 1H), 7.61 (dd, *J* = 8.9, 2.5 Hz, 1H), 7.68 (d, *J* = 2.5 Hz, 1H), 9.80 (s, 1 H), 10.90 (s, 1H) ppm. **^{13}C{1H} NMR** (125 MHz, $CDCl_3$): δ = 195.4, 160.6, 139.7, 135.6, 121.8, 119.8, 111.4 ppm. **IR** (ATR): λ^{-1} = 3224, 1670, 1651, 1610, 1562, 1464, 1425, 1372, 1270, 1152, 1114, 1070, 891, 827, 765, 691, 627, 537. **GC/MS**: 201 (97), 199 (100), 182 (10), 173 (12), 154 (14), 117 (5), 92 (9).

The spectroscopic values are in accordance with literature values.[5]

4-Bromo-*N,N*-dimethylaniline (6j)

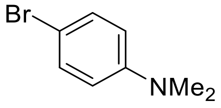

According to the General Procedure, *N,N*-dimethylaniline (0.50 mmol, 1.0 equiv.) and tetrabutylammonium bromide (645 mg, 2.00 mmol, 4.0 equiv.) were converted to furnish product **6j** as a white solid (104 mg, 0.465 mmol, 93%, 155% CE) after 1.2 *F* (96 min, 10 mA). After complete consumption of the starting material, the reaction mixture was neutralized with sodium bicarbonate and then extracted with n-pentane (3 × 20 mL).

^{1}H NMR (500 MHz, $CDCl_3$): δ = 2.83 (s, 6H), 6.50 (d, *J* = 9.1 Hz, 2H), 7.21 (d, *J* = 9.1 Hz, 2H) ppm. **^{13}C{1H} NMR** (125 MHz, $CDCl_3$): δ = 149.6, 131.7, 114.1, 108.5, 40.5 ppm. **IR** (ATR): λ^{-1} = 2983, 2885, 2853, 2801, 1585, 1492, 1444, 1347, 1221, 1188, 1122, 1075, 1062, 988, 942, 802, 750, 694, 578. **GC/MS**: 201 (95), 199 (100), 183 (15), 157 (11), 118 (59), 104 (29), 91(16), 77 (41), 63 (26).

The spectroscopic values are in accordance with literature values.[10]

2-Bromo-*N,N*,4-trimethylaniline (6k)

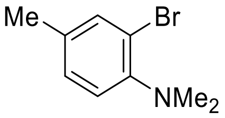

According to the General Procedure, *N*,*N*,4-trimethylaniline (0.50 mmol, 1.0 equiv.) and tetrabutylammonium bromide (645 mg, 2.00 mmol, 4.0 equiv.) were converted to furnish product **6k** as a dark yellow oil (103 mg, 0.485 mmol, 97%, 162% CE) after 1.2 *F* (96 min, 10 mA). After complete consumption of the starting material, the reaction mixture was neutralized with sodium bicarbonate and then extracted with n-pentane (3 × 20 mL).

^{1}**H NMR** (500 MHz, $CDCl_3$): δ = 2.19 (s, 3H), 2.68 (s, 6H), 6.90 (d, *J* = 8.2 Hz, 1H), 6.97 (dd, *J* = 7.9, 2.0 Hz, 1H), 7.30 (d, *J* = 2.2 Hz, 1H) ppm. ^{13}**C{1H} NMR** (125 MHz, $CDCl_3$): δ = 149.4, 134.3, 133.9, 128.7, 120.3, 119.2, 44.4, 20.2 ppm. **GC/MS**: 216 (8), 214 (100), 198 (8), 169 (3), 132 (55), 91 (58), 65 (31).

The spectroscopic values are in accordance with literature values.[11]

2-Bromo-4-methylaniline (6l)

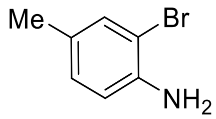

According to the General Procedure, *p*-toluidine (0.50 mmol, 1.0 equiv.) and tetrabutylammonium bromide (645 mg, 2.00 mmol, 4.0 equiv.) were converted to furnish product **6l** as a brown oil (87 mg, 0.47 mmol, 94%, 157% CE) after 1.2 *F* (96 min, 10 mA). After complete consumption of the starting material, the reaction mixture was neutralized with sodium bicarbonate and then extracted with n-pentane (3 × 20 mL).

^{1}**H NMR** (500 MHz, $CDCl_3$): δ = 2.13 (s, 3H), 3.82 (broad, 2H), 6.57 (d, *J* = 8.1 Hz, 1H), 6.81 (dd, *J* = 8.1, 2.0 Hz, 1H), 7.14 (d, *J* = 1.9 Hz, 1H) ppm. ^{13}**C{1H} NMR** (125 MHz, $CDCl_3$): δ = 141.6, 132.8, 129.1, 129.0, 115.8, 109.3, 20.1 ppm. **GC/MS**: 187 (39), 185 (37), 106 (100), 89 (3), 77 (41).

The spectroscopic values are in accordance with literature values.[3]

2-Bromo-4-(trifluoromethyl)aniline (6m)

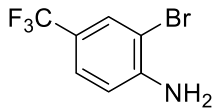

According to the General Procedure, 4-(trifluoromethyl)aniline (0.50 mmol, 1.0 equiv.) and tetrabutylammonium bromide (645 mg, 2.00 mmol, 4.0 equiv.) were converted to furnish product **6m** as a white solid (111 mg, 0.465 mmol, 93%, 143% CE) after 1.3 *F* (104 min, 10 mA). After complete consumption of the starting material, the reaction mixture was neutralized with sodium bicarbonate and then extracted with n-pentane (3 × 20 mL).

^{1}**H NMR** (500 MHz, $CDCl_3$): δ = 4.42 (s, 2H), 6.80 (dd, *J* = 14.3, 8.3 Hz, 1H), 7.42-7.30 (m, 1H), 7.71 (d, *J* = 13.2 Hz, 1H) ppm. ^{13}**C{1H} NMR** (125 MHz, $CDCl_3$): δ = 147.0, 129.9 (q, *J* = 3.8 Hz), 128.7 (q, *J* = 270.0 Hz), 121.0 (q, *J* = 33.7 Hz), 114.7, 108.1 ppm. **IR** (ATR): λ^{-1} = 3425, 3311, 3067, 1624, 1604, 1508, 1415, 1318, 1301, 1264, 1138, 1095, 1071, 1035, 891, 824, 681, 617. **GC/MS**: 241 (100), 239 (100), 220 (100), 189 (3), 160 (41), 140 (36), 113 (25).

The spectroscopic values are in accordance with literature values.[4]

4-Bromo-1*H*-pyrazole (6n)

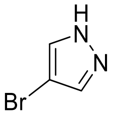

According to the General Procedure, 1*H*-pyrazole (0.50 mmol, 1.0 equiv.) and tetrabutylammonium bromide (645 mg, 2.00 mmol, 4.0 equiv.) were converted to furnish product **6n** as a white solid (72 mg, 0.495 mmol, 99%, 174% CE) after 1.15 *F* (92 min, 10 mA).

^{1}H NMR (500 MHz, DMSO-d_6): δ = 7.77 (s, 2H), 12.20 (broad, 1H) ppm. **^{13}C{1H} NMR** (125 MHz, DMSO-d_6): δ = 134.3, 92.2 ppm. **IR** (ATR): λ^{-1} = 3138, 3073, 2850, 2363, 1510, 1444, 1387, 1239, 1193, 1163, 1126, 1077, 1053, 943, 911, 846, 714, 623, 597. **GC/MS**: 148 (100), 146 (99), 119 (26), 119 (2), 92 (18), 67 (19).

The spectroscopic values are in accordance with literature values.[15]

4-Bromo-3,5-dimethyl-1*H*-pyrazole (6o)

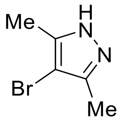

According to the General Procedure, 3,5-dimethyl-1*H*-pyrazole (0.50 mmol, 1.0 equiv.) and tetrabutylammonium bromide (645 mg, 2.00 mmol, 4.0 equiv.) were converted to furnish product **6o** as a white solid (86 mg, 0.495mmol, 99%, 182% CE) after 1.1 *F* (88 min, 10 mA).

^{1}H NMR (500 MHz, $CDCl_3$): δ = 2.18 (s, 6H), 10.17 (very broad, <1H) ppm. **^{13}C{1H} NMR** (125 MHz, $CDCl_3$): δ = 142.8, 94.2, 11.3 ppm. **IR** (ATR): λ^{-1} = 3176, 3099, 3039, 2970, 2864, 2360, 1739, 1580, 1471, 1413, 1377, 1301, 1260, 1229, 1217,1149, 1094, 1041, 1000, 809, 764, 669, 629, 599. **GC/MS**: 176 (86), 174 (85), 159 (2), 119 (2), 95 (100), 65 (46), 54 (43).

The spectroscopic values are in accordance with literature values.[14] However, we seem to be the first to detect the NH-proton in ^{1}H NMR for this compound.

1,3-Dibromoazulene (6p)

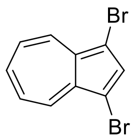

According to the General Procedure, azulene (0.50 mmol, 1.0 equiv.) and tetrabutylammonium bromide (645 mg, 2.00 mmol, 4.0 equiv.) were converted to furnish product **6p** as a white solid (142 mg, 0.495 mmol, 99%, 154% CE) after 2.6 *F* (209 min, 10 mA).

^{1}H NMR (500 MHz, DMSO-d_6): δ = 7.15 (t, *J* = 9.8 Hz, 2H), 7.55 (t, *J* = 9.9 Hz, 1H), 7.69 (s, 1H), 8.18 (d, *J* = 9.2 Hz, 2H) ppm. **^{13}C{1H} NMR** (125 MHz, DMSO-d_6): δ = 140.0, 138.2, 136.7, 135.8, 124.0, 102.7 ppm. **IR** (ATR): λ^{-1} = 1575, 1480, 1381, 1290, 1217, 1145, 1087,932, 880, 867, 721, 591, 561. **GC/MS**: 286(70), 284 (35.4), 260 (6.5), 207 (6.4), 126(100), 98 (14.2), 63 (25.5).

The spectroscopic values are in accordance with literature values.[16]

5,7-Dibromoquinolin-8-ol (6q)

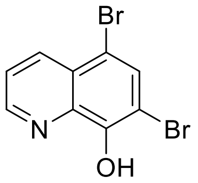

According to the General Procedure, quinolin-8-ol (0.50 mmol, 1.0 equiv.) and tetrabutylammonium bromide (645 mg, 2.00 mmol, 4.0 equiv.) were converted to furnish product **6q** as a white solid (111 mg, 0.37 mmol, 74%, 129% CE) after 2.3 *F* (185 min, 10 mA).

^{1}H NMR (500 MHz, DMSO-d_6): δ = 7.78 (dd, J = 8.5, 4.2 Hz, 1H), 8.06 (s, 1H), 8.45 (dd, J = 8.5, 1.5 Hz, 1H), 8.97 (dd, J = 4.2, 1.5 Hz, 1H), ppm. **^{13}C{1H} NMR** (125 MHz, DMSO-d_6): δ = 151.6, 150.3, 139.4, 135.9, 133.8, 127.0, 124.2, 109.3, 105.6 ppm. **GC/MS**: 305 (43), 303 (100), 301 (53), 196 (79), 57 (36).

The spectroscopic values are in accordance with literature values.[3]

3.2. NMR spectra of all synthesized compounds

1-Bromo-4-methoxybenzene (6a)

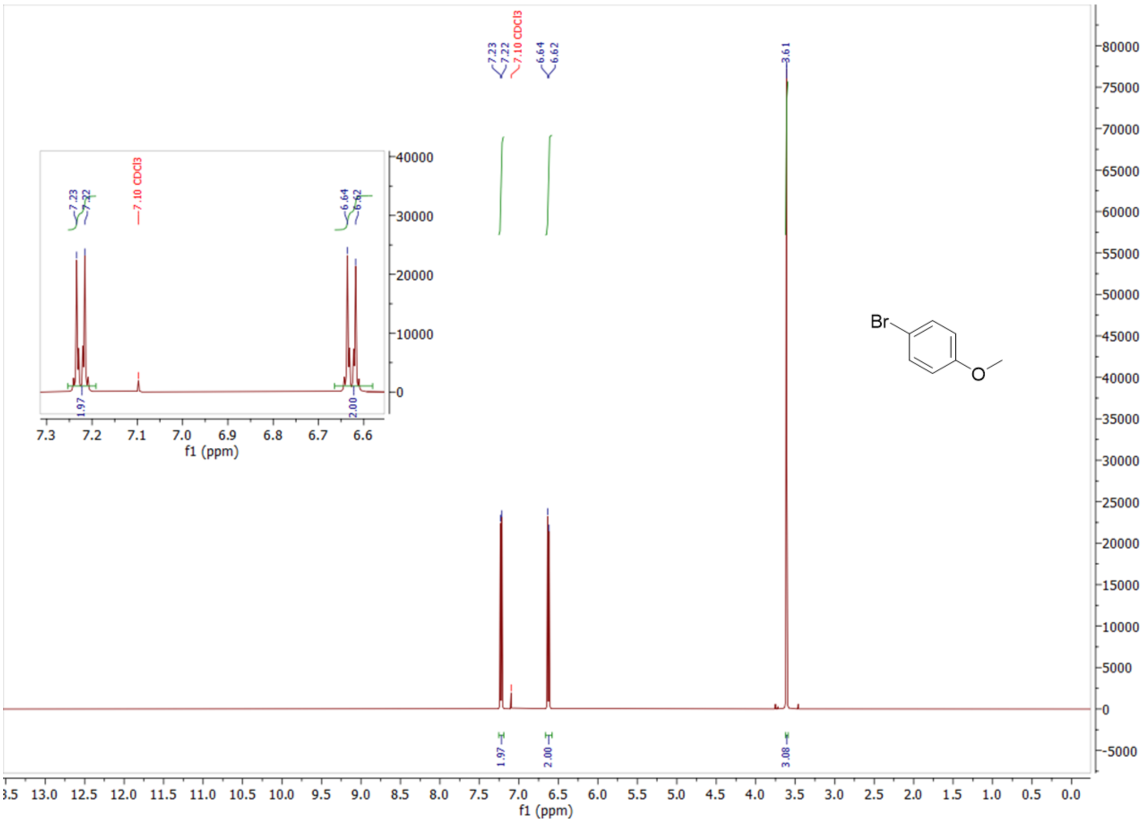

Figure S2: ^{1}H NMR (500 MHz, $CDCl_3$) of compound **6a**.

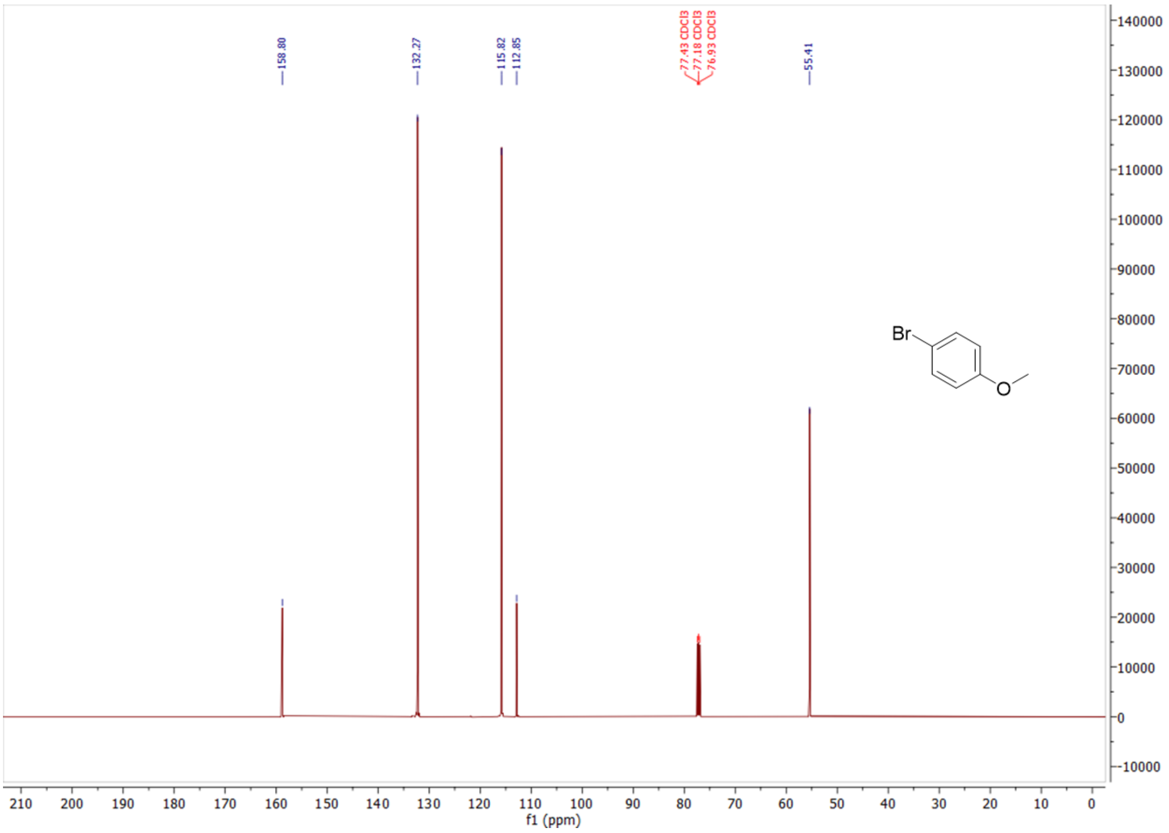

Figure S3: $^{13}C\{^1H\}$ NMR (125 MHz, $CDCl_3$) of compound **6a**.

1-Bromo-2-methoxynaphthalene (6b)

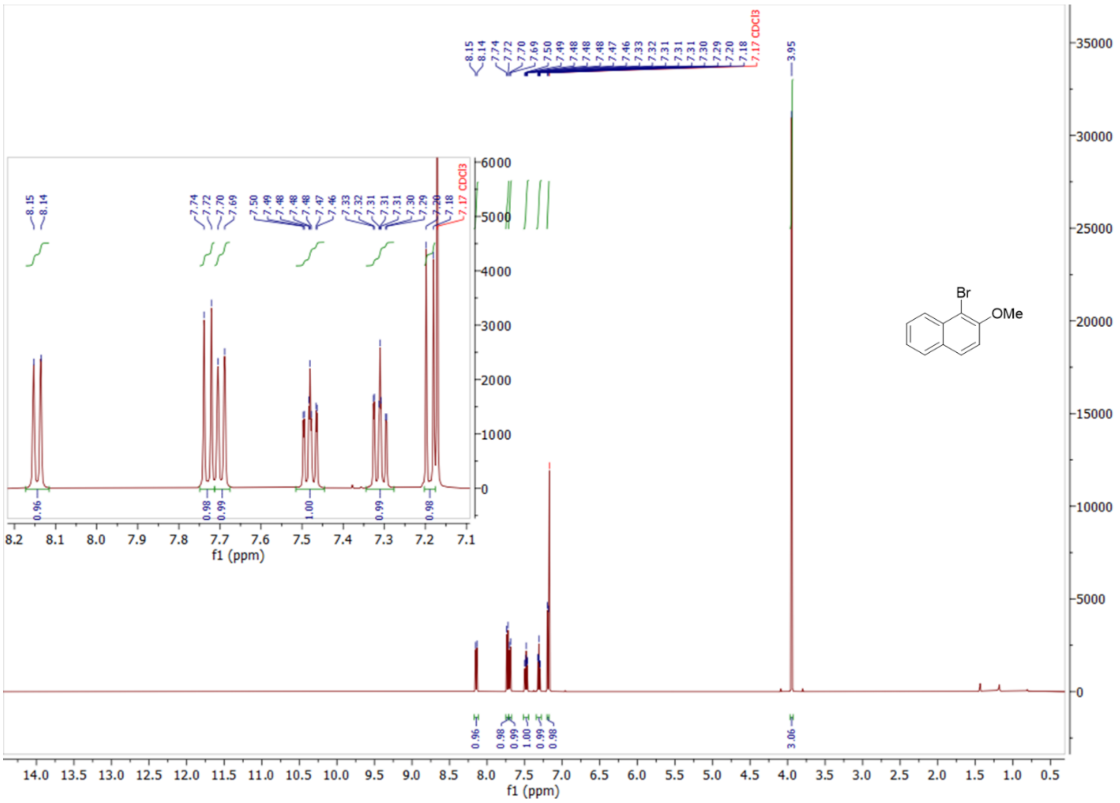

Figure S4: ^1H NMR (500 MHz, $CDCl_3$) of compound **6b**.

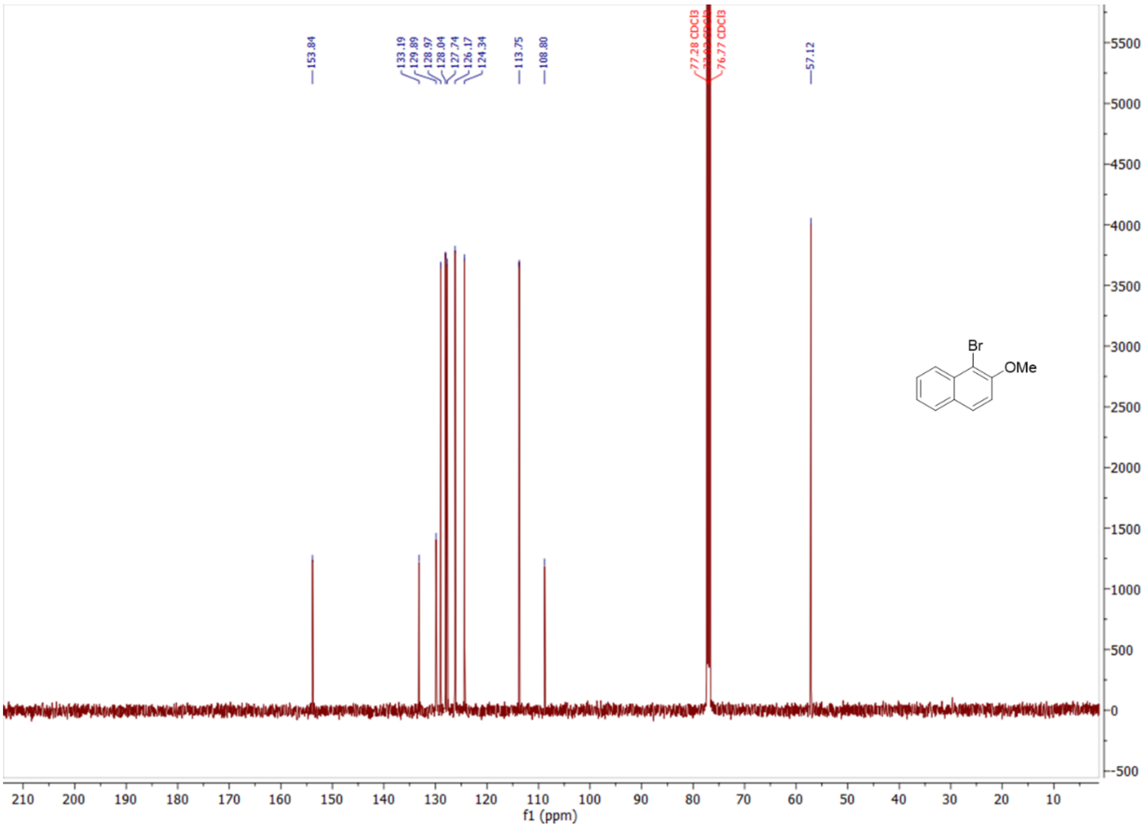

Figure S5: $^{13}C\{1H\}$ NMR (125 MHz, $CDCl_3$) of compound **6b**.

4-Bromo-1,2-dimethoxybenzene (6c)

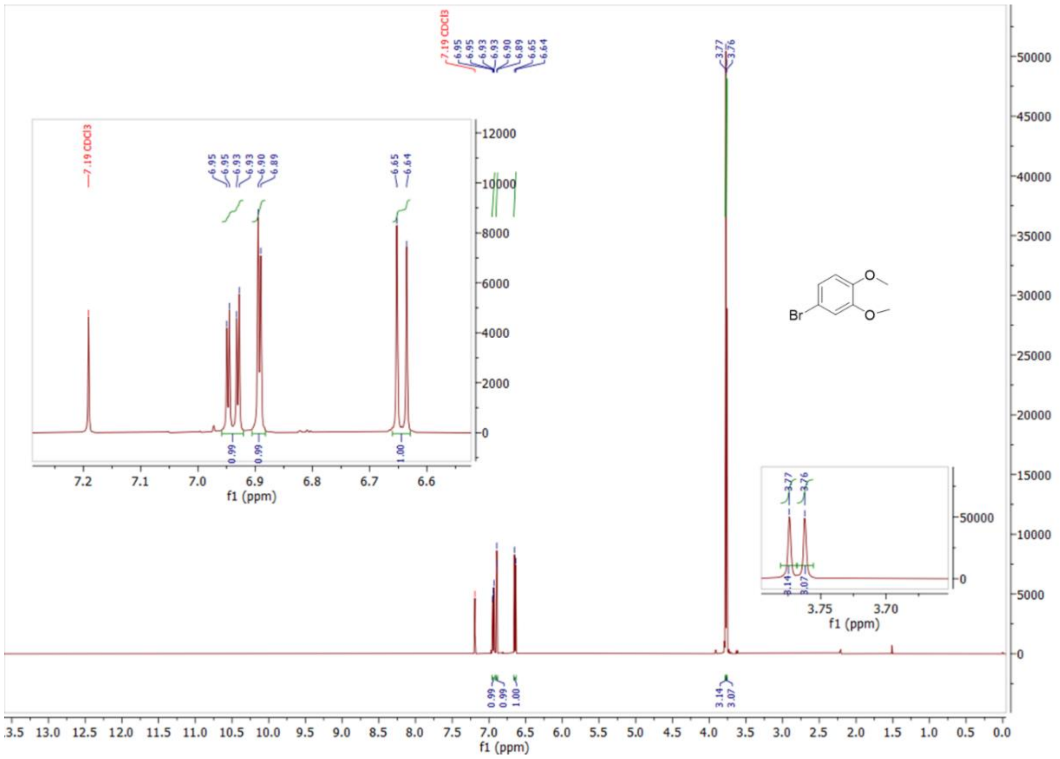

Figure S6: ^{1}H NMR (500 MHz, $CDCl_3$) of compound **6c**.

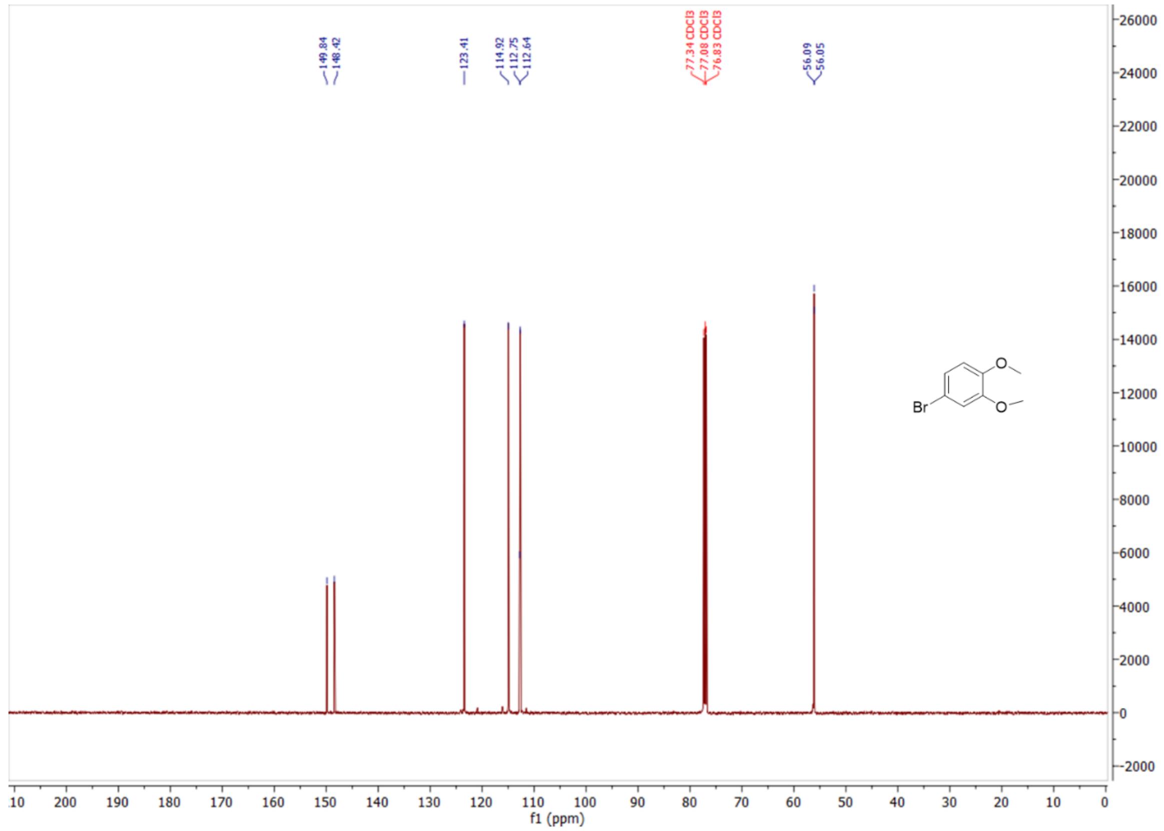

Figure S7: $^{13}C\{1H\}$ NMR (125 MHz, $CDCl_3$) of compound **6c**.

1,2-Dibromo-4,5-dimethoxybenzene (**6d**)

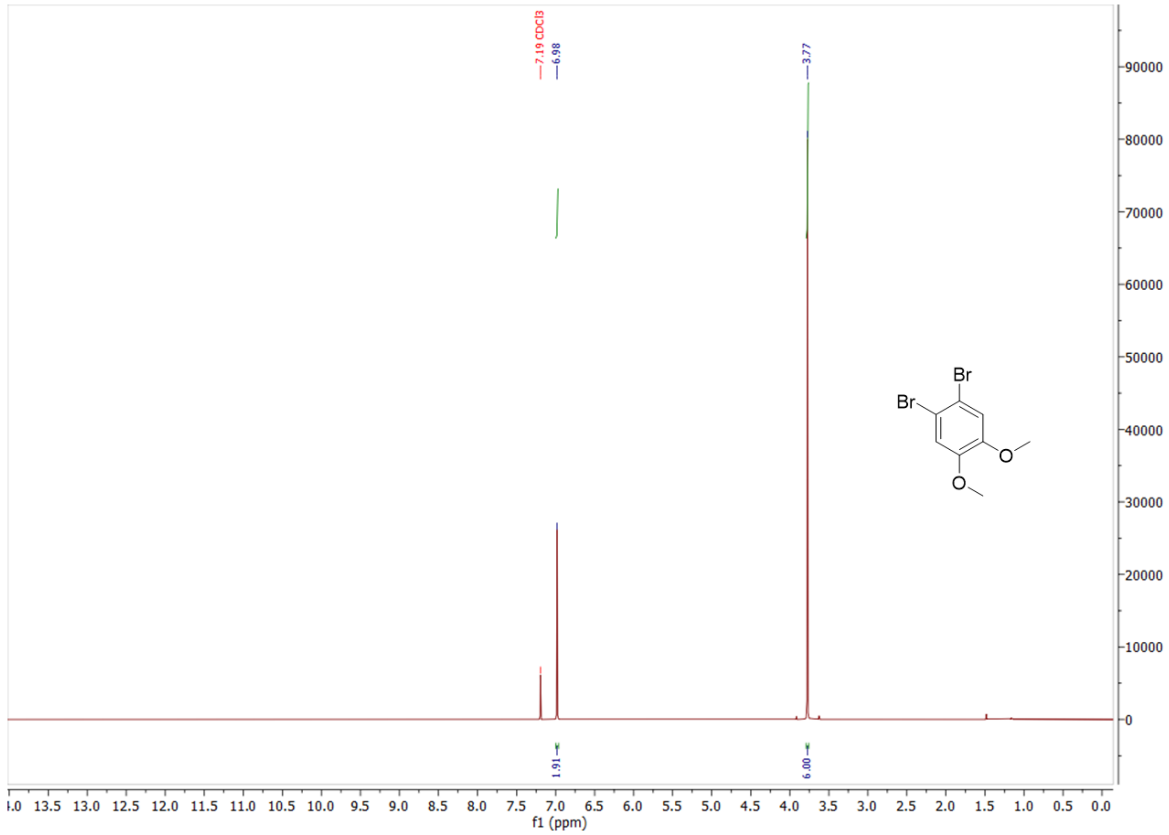

Figure S8: ^{1}H NMR (500 MHz, $CDCl_3$) of compound **6d**.

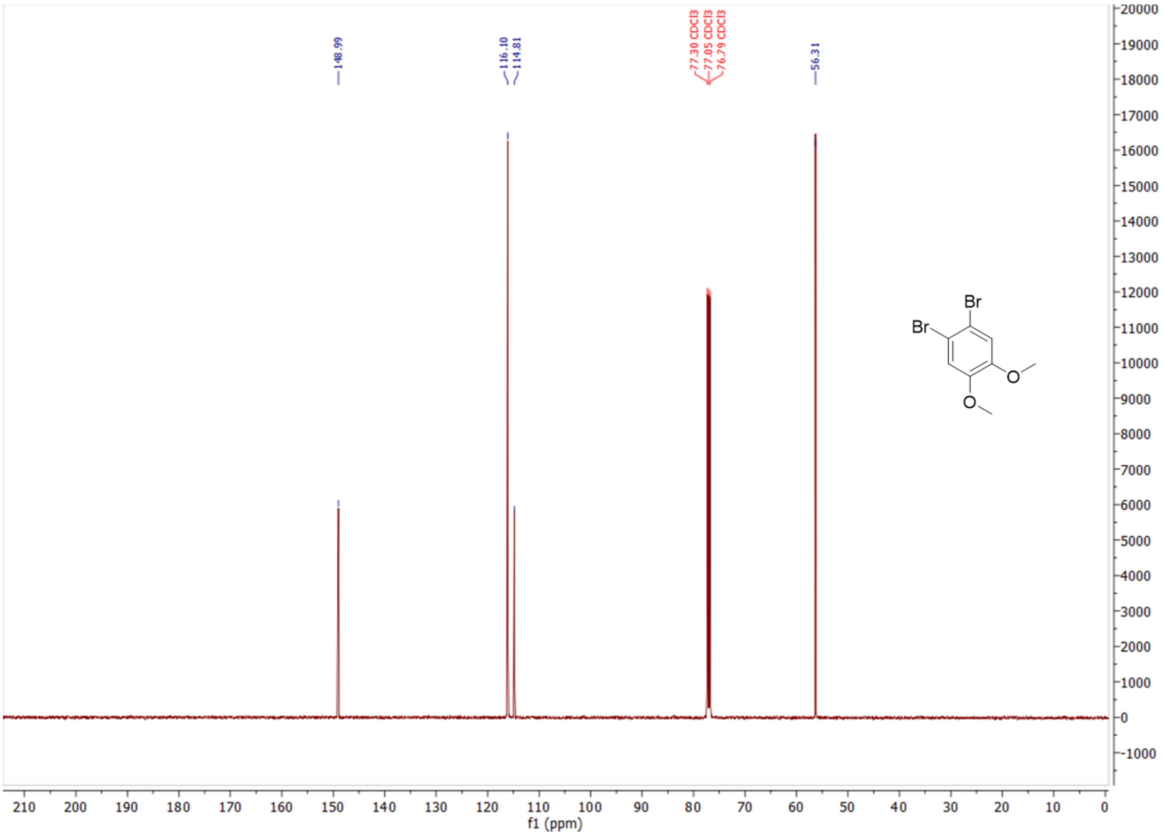

Figure S9: $^{13}C\{^1H\}$ NMR (125 MHz, $CDCl_3$) of compound **6d**.

1-Bromo-4-methoxy-2-methylbenzene (6e)

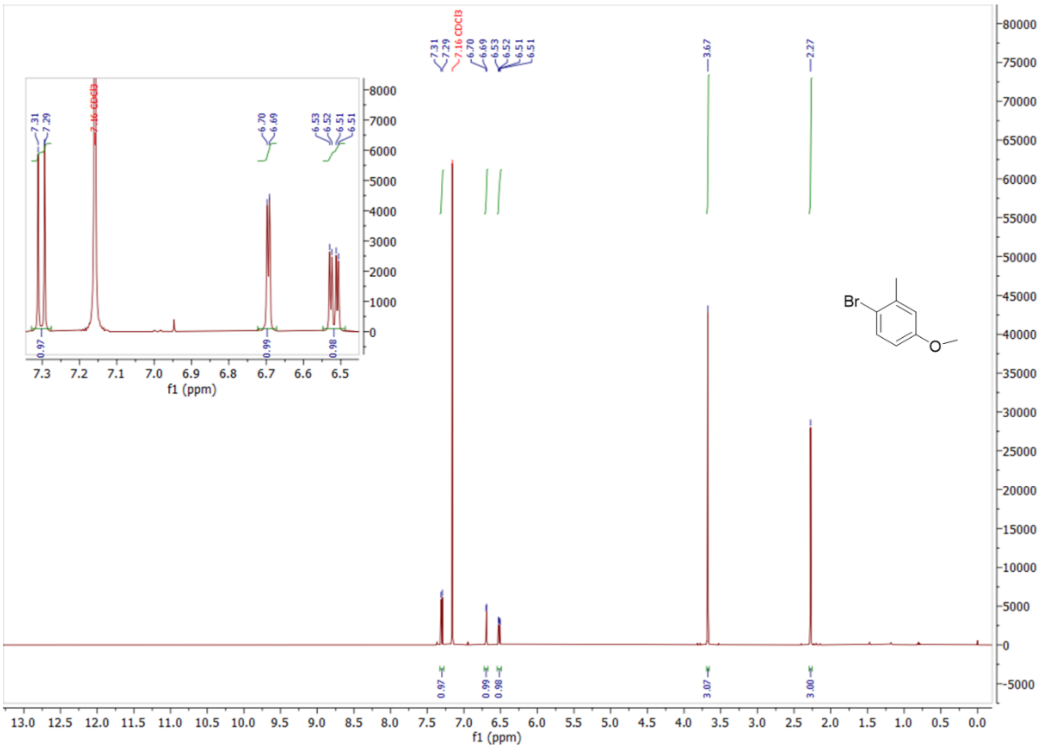

Figure S10: ^1H NMR (500 MHz, $CDCl_3$) of compound **6e**.

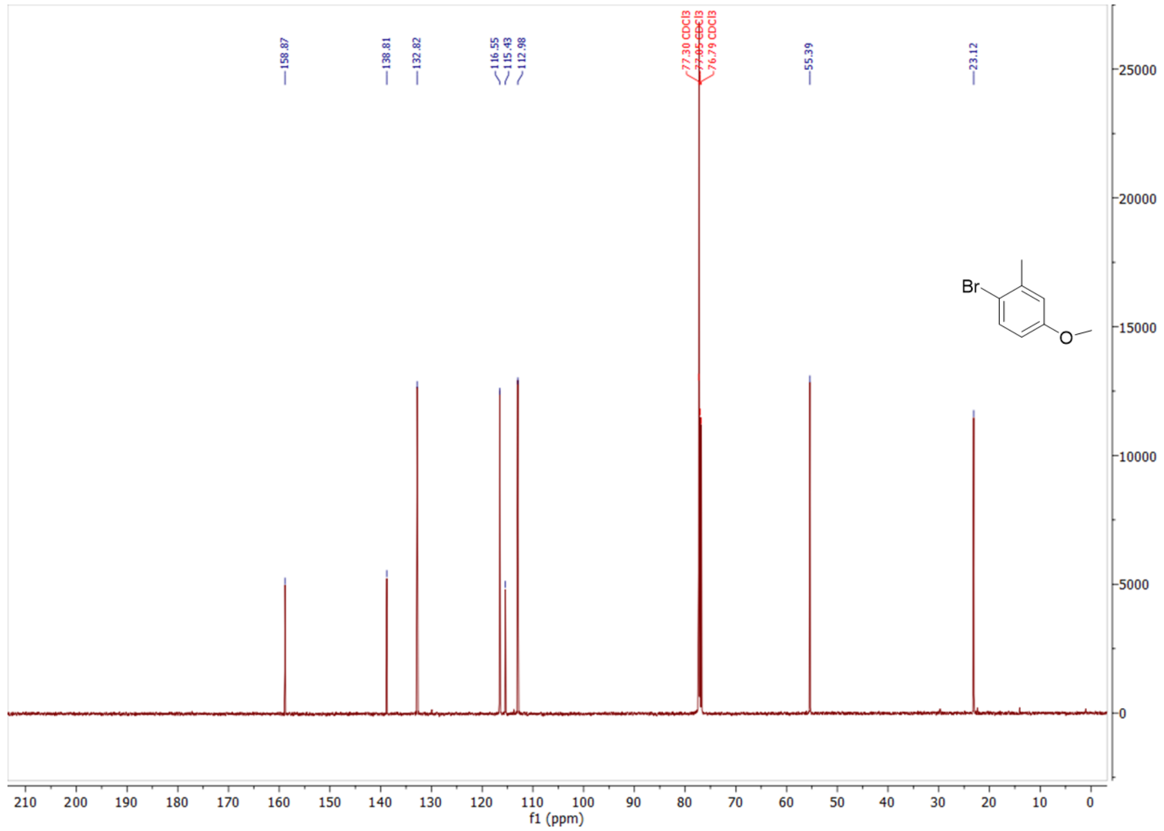

Figure S11: $^{13}C\{1H\}$ NMR (125 MHz, $CDCl_3$) of compound **6e**.

2-Bromo-5-methoxy-1,3-dimethylbenzene (6f)

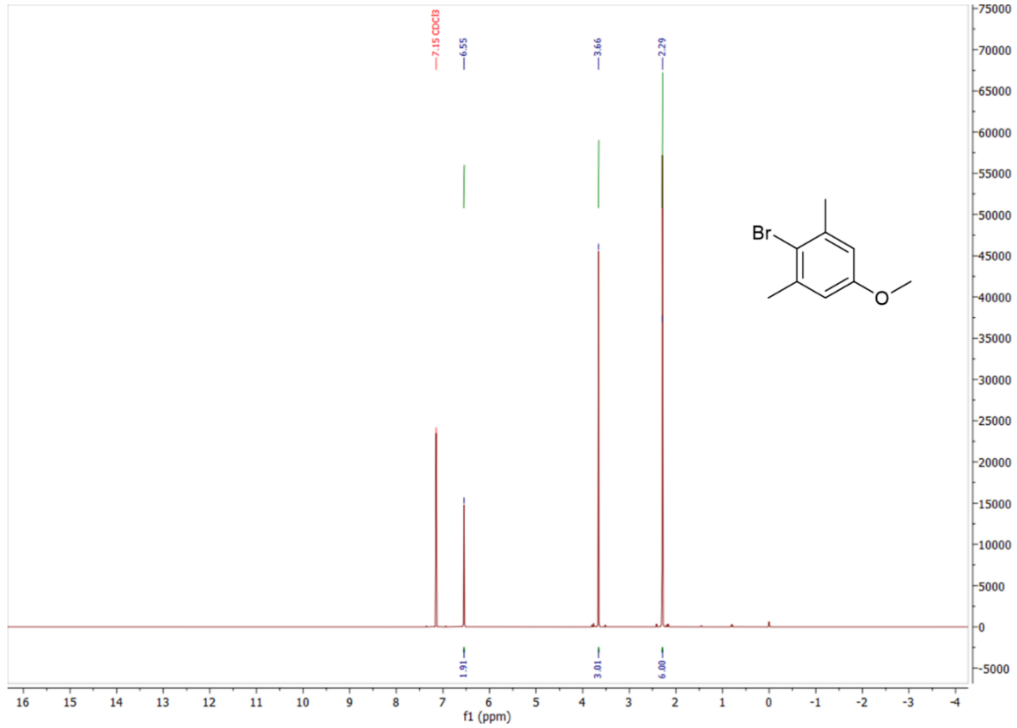

Figure S12: ^{1}H NMR (500 MHz, $CDCl_3$) of compound **6f**.

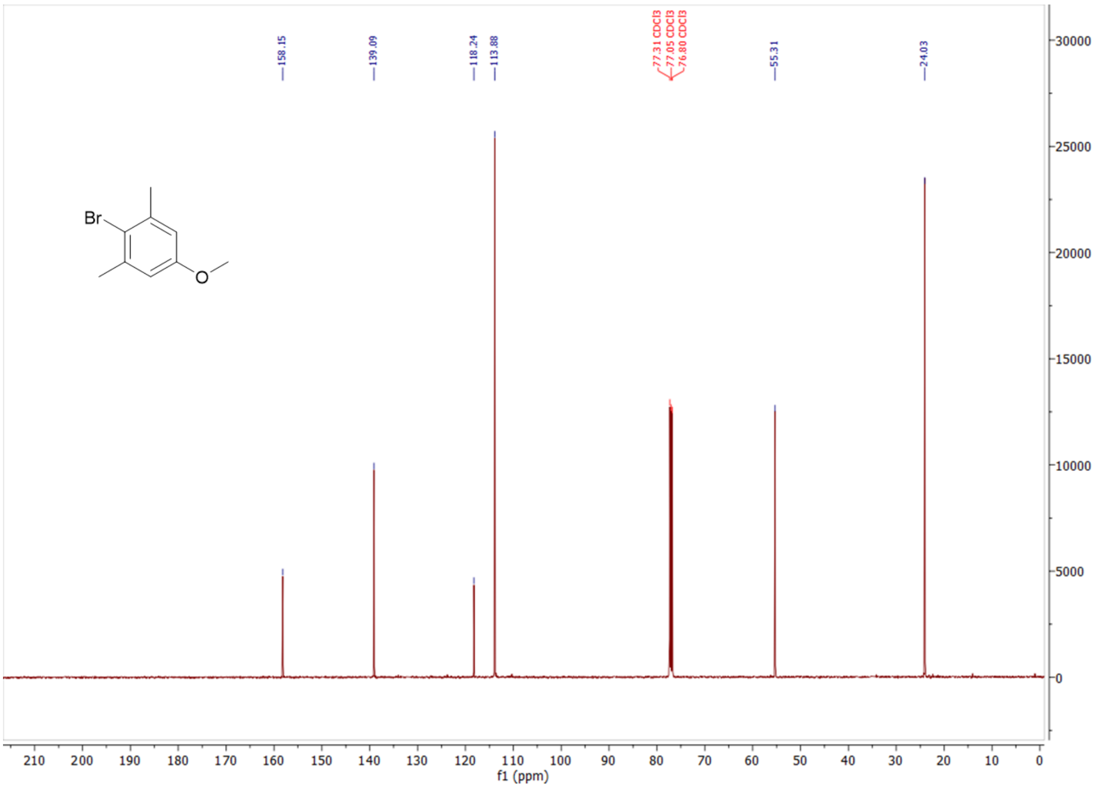

Figure S13: $^{13}C\{1H\}$ NMR (125 MHz, $CDCl_3$) of compound **6f**.

4-Bromophenol (6g)

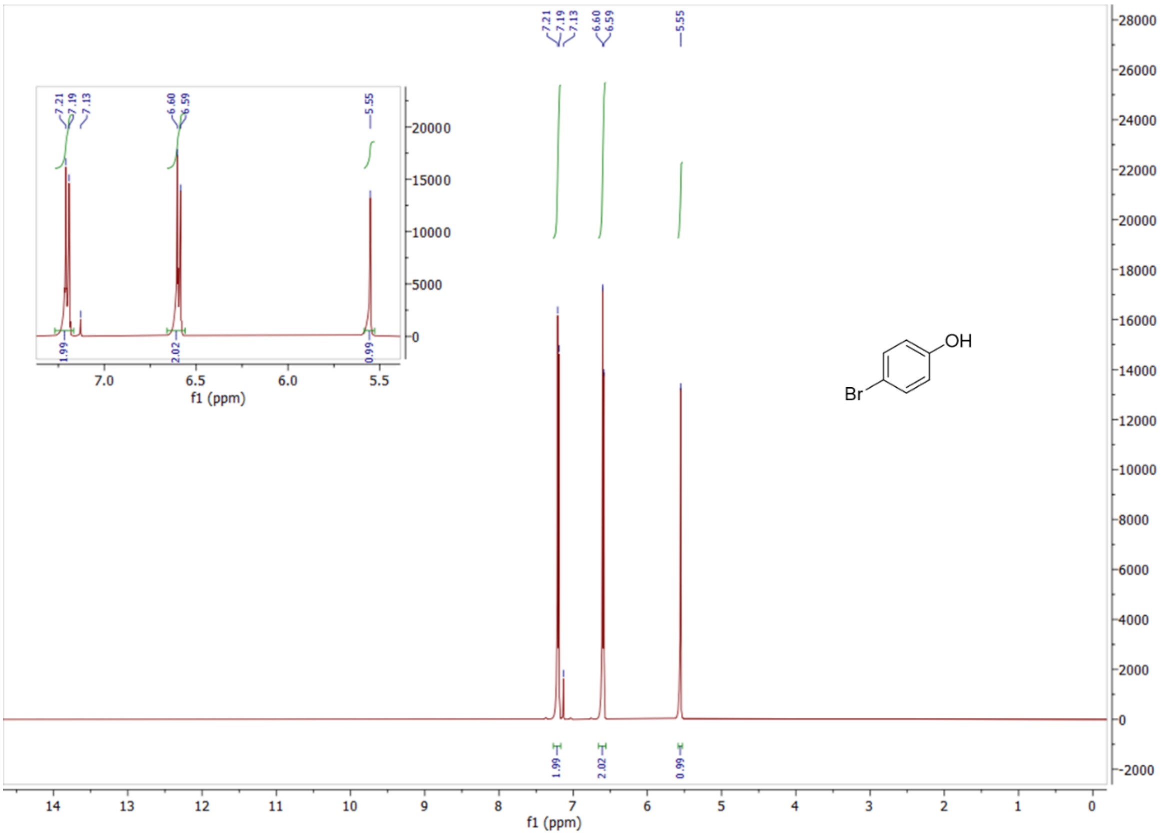

Figure S14: ^{1}H NMR (500 MHz, $CDCl_3$) of compound **6g**.

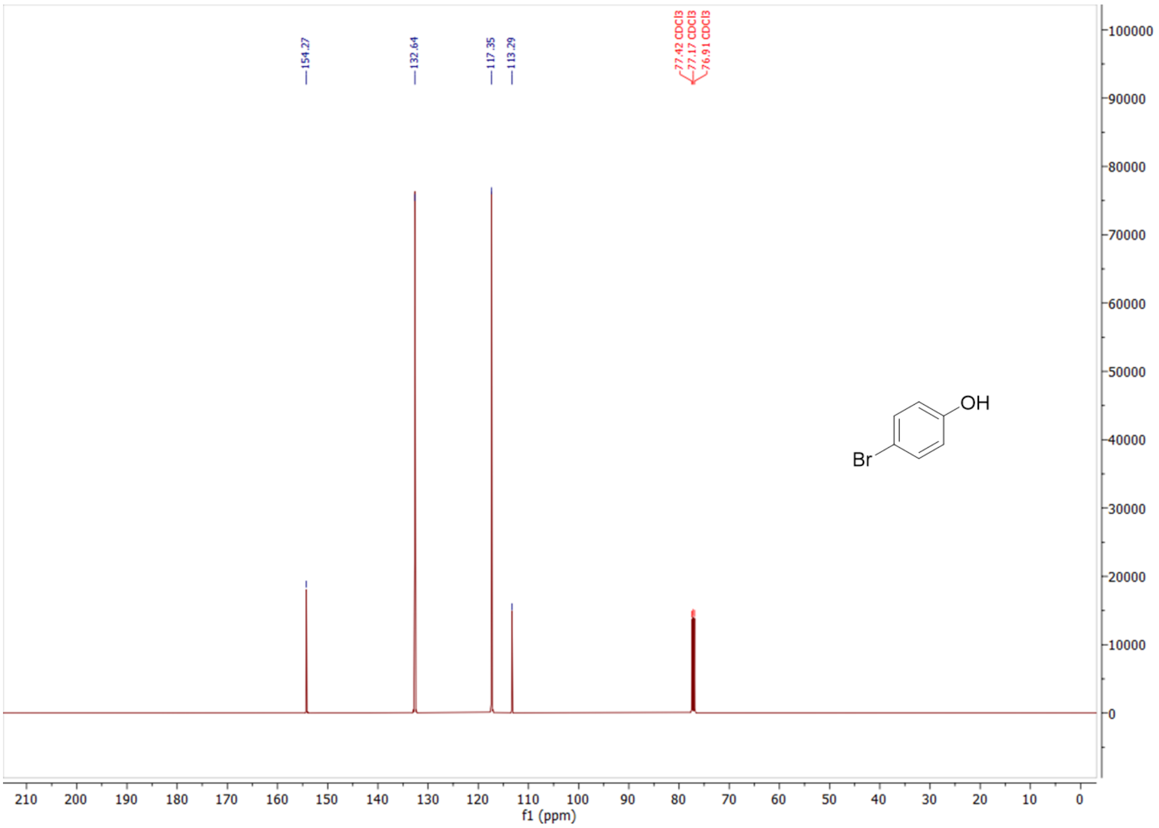

Figure S15: $^{13}C\{1H\}$ NMR (125 MHz, $CDCl_3$) of compound **6g**.

2-Bromo-4-(*tert-butyl*)phenol (6h)

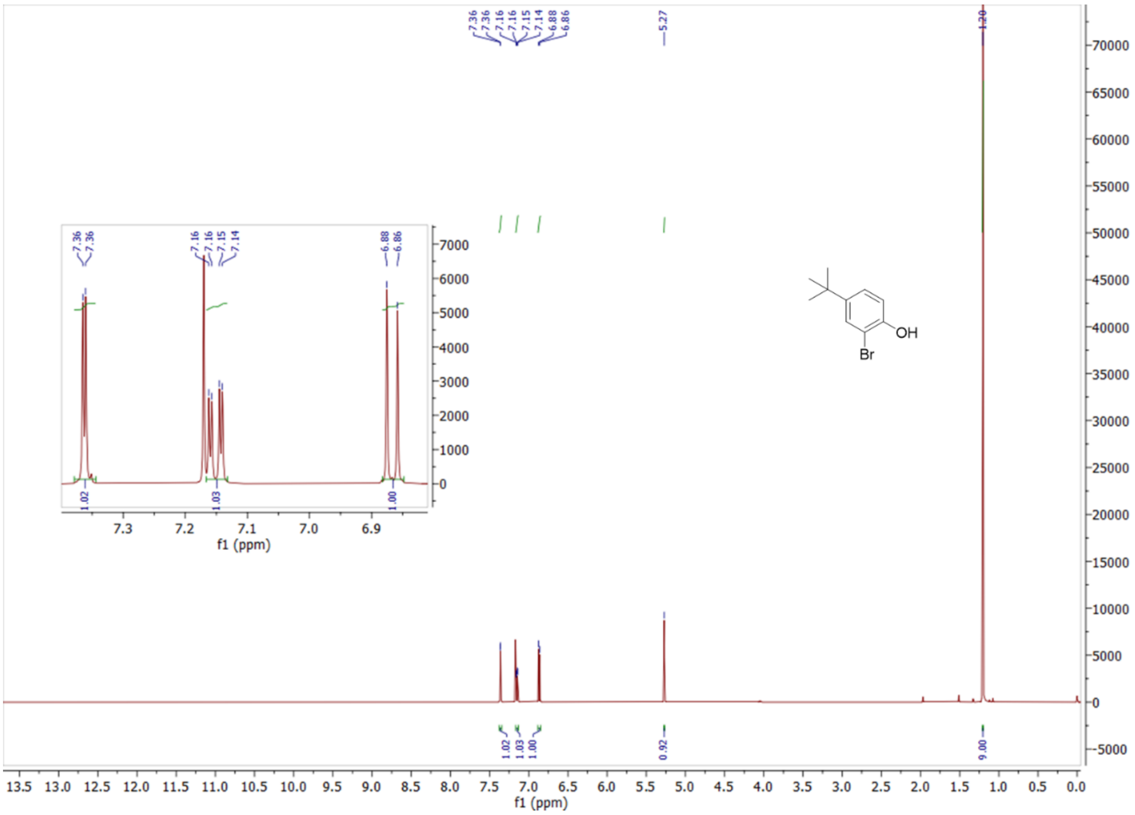

Figure S16: ^{1}H NMR (500 MHz, $CDCl_3$) of compound **6h**.

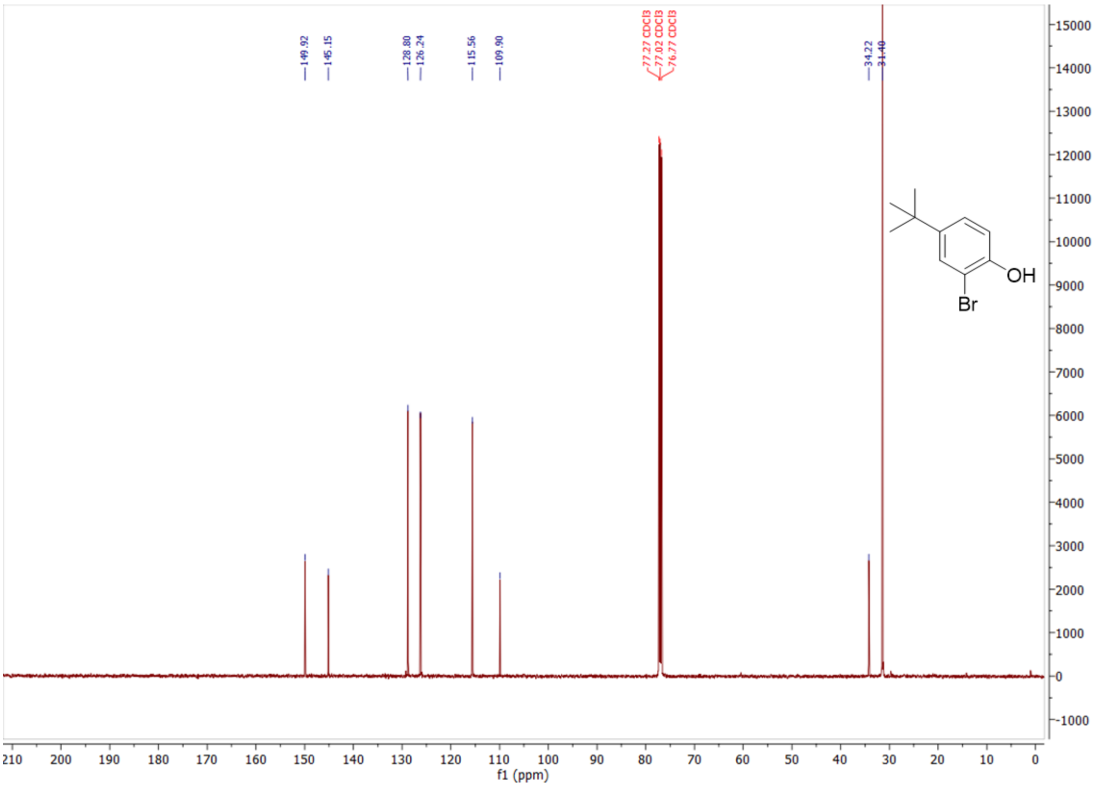

Figure S17: $^{13}C\{1H\}$ NMR (125 MHz, $CDCl_3$) of compound **6h**.

5-Bromo-2-hydroxybenzaldehyde (6i)

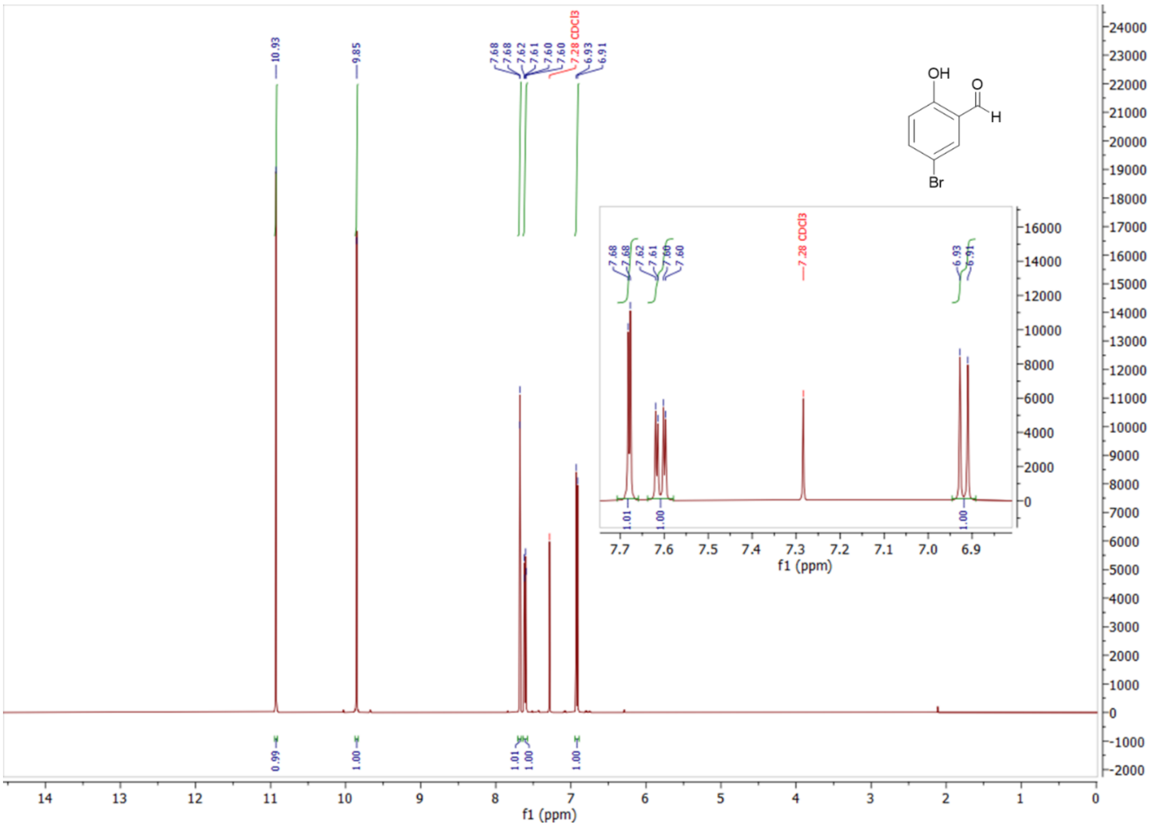

Figure S18: ^{1}H NMR (500 MHz, $CDCl_3$) of compound **6i**.

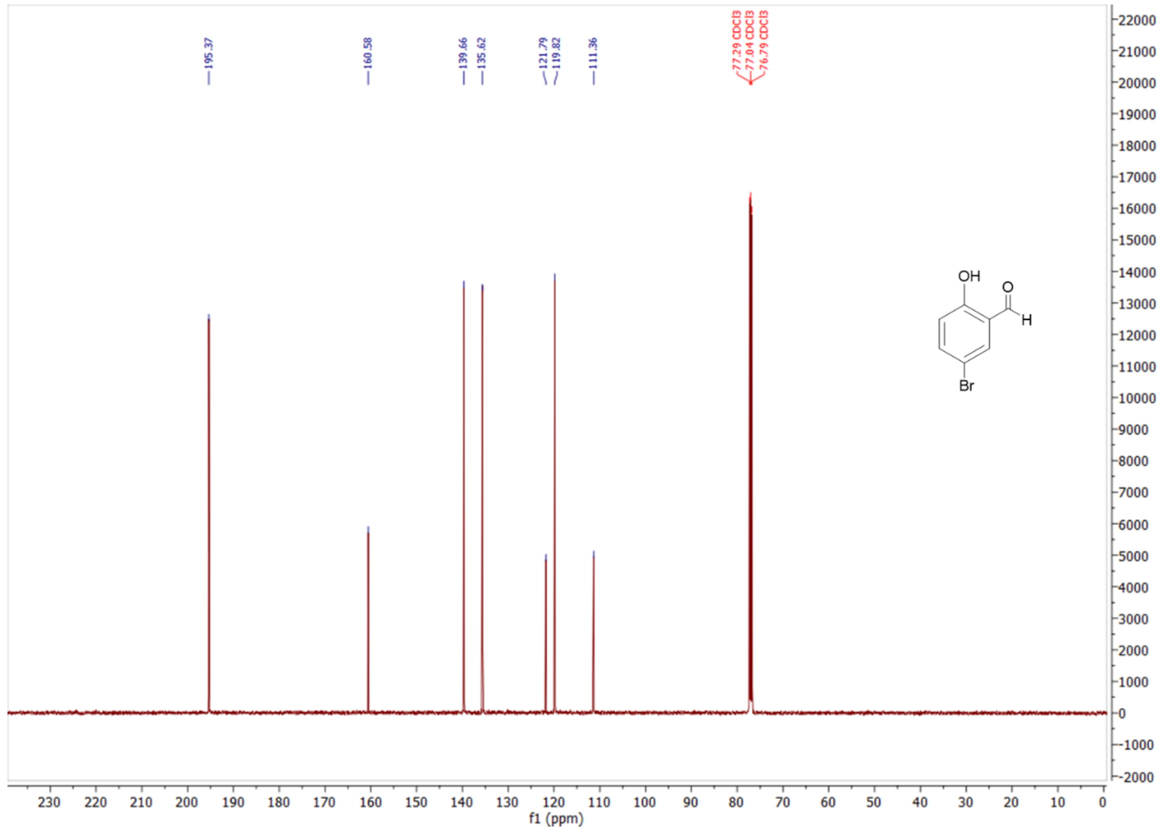

Figure S19: $^{13}C\{1H\}$ NMR (125 MHz, $CDCl_3$) of compound **6i**.

4-Bromo-*N*,*N*-dimethylaniline (6j)

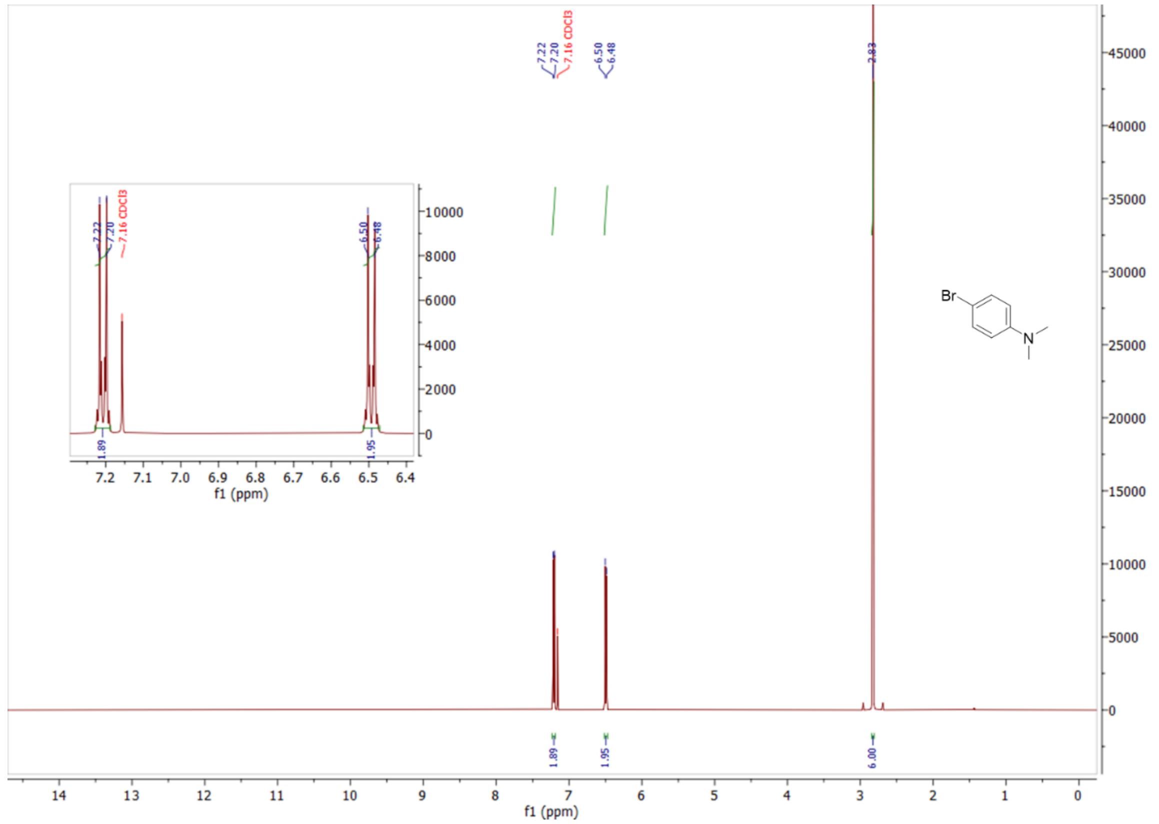

Figure S20: ^{1}H NMR (500 MHz, $CDCl_3$) of compound **6j**.

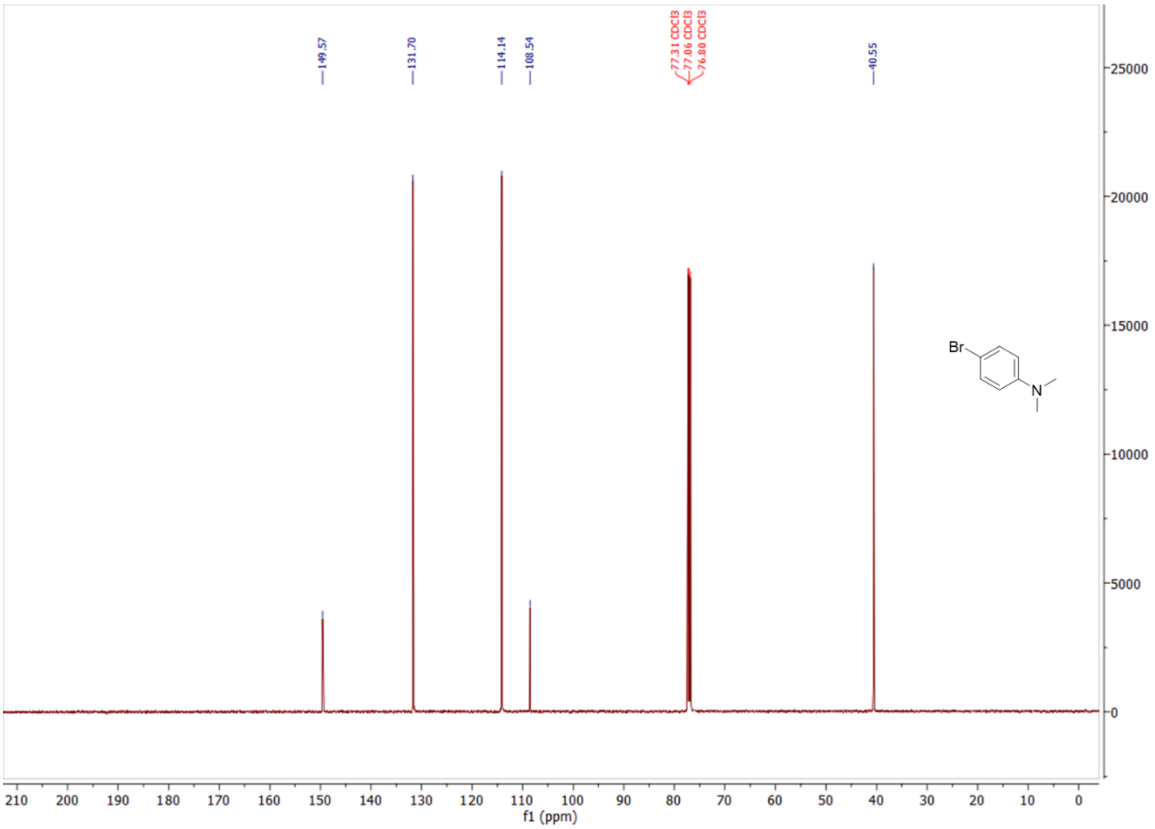

Figure S21: ^{13}C{1H} NMR (125 MHz, $CDCl_3$) of compound **6j**.

2-Bromo-*N*,*N*,4-trimethylaniline (6k)

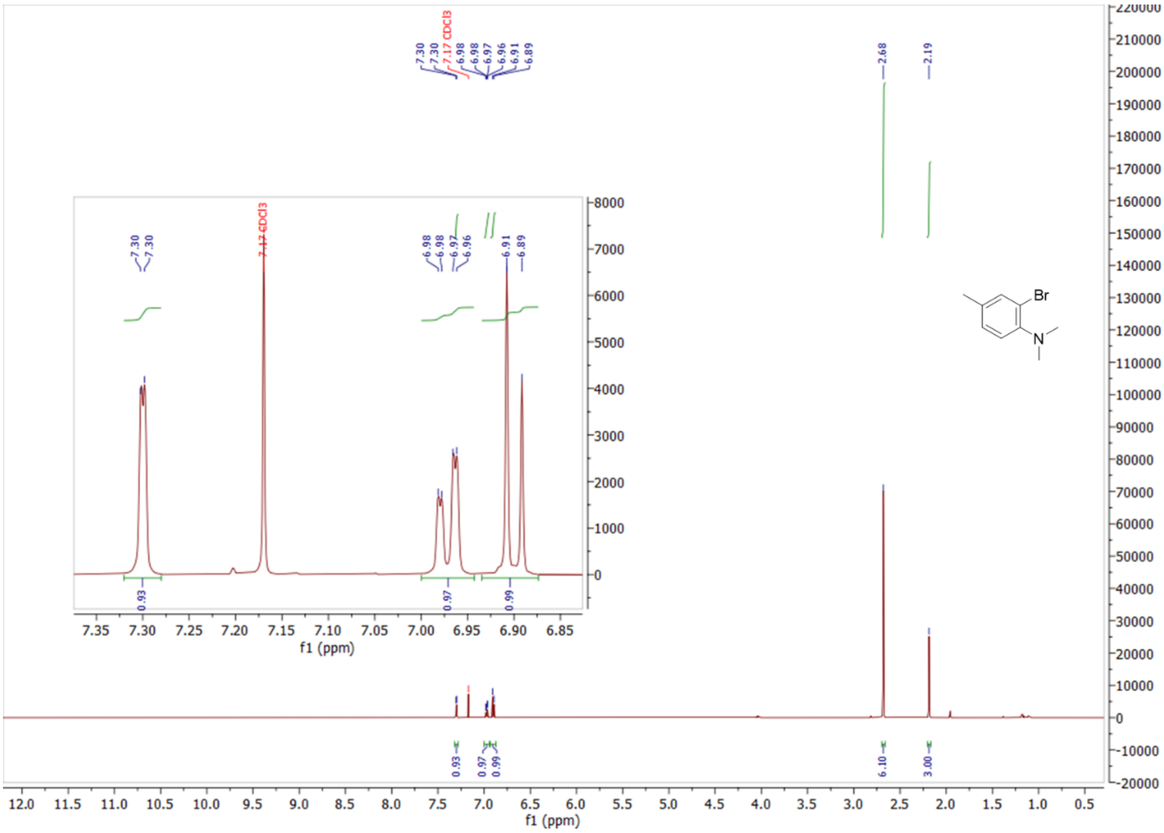

Figure S22: ^{1}H NMR (500 MHz, $CDCl_3$) of compound **6k**.

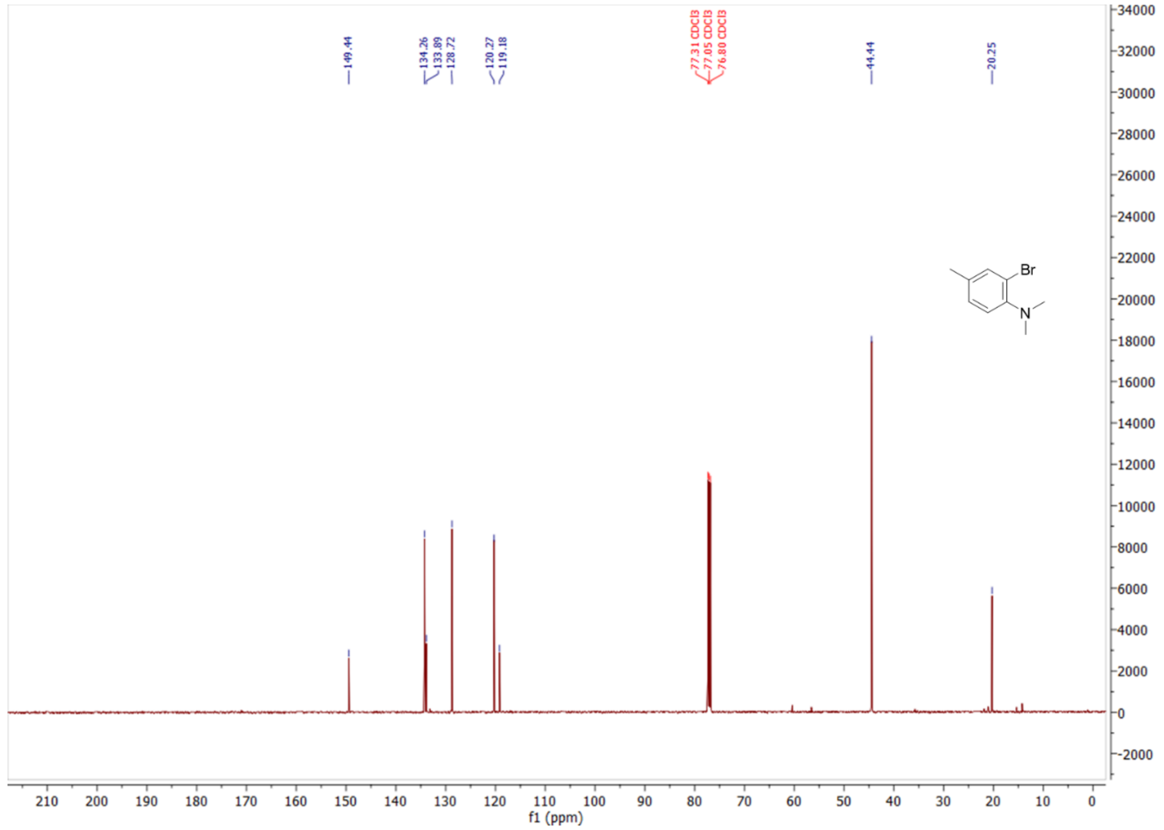

Figure S23: $^{13}C\{1H\}$ NMR (125 MHz, $CDCl_3$) of compound **6k**.

2-Bromo-4-methylaniline (6l)

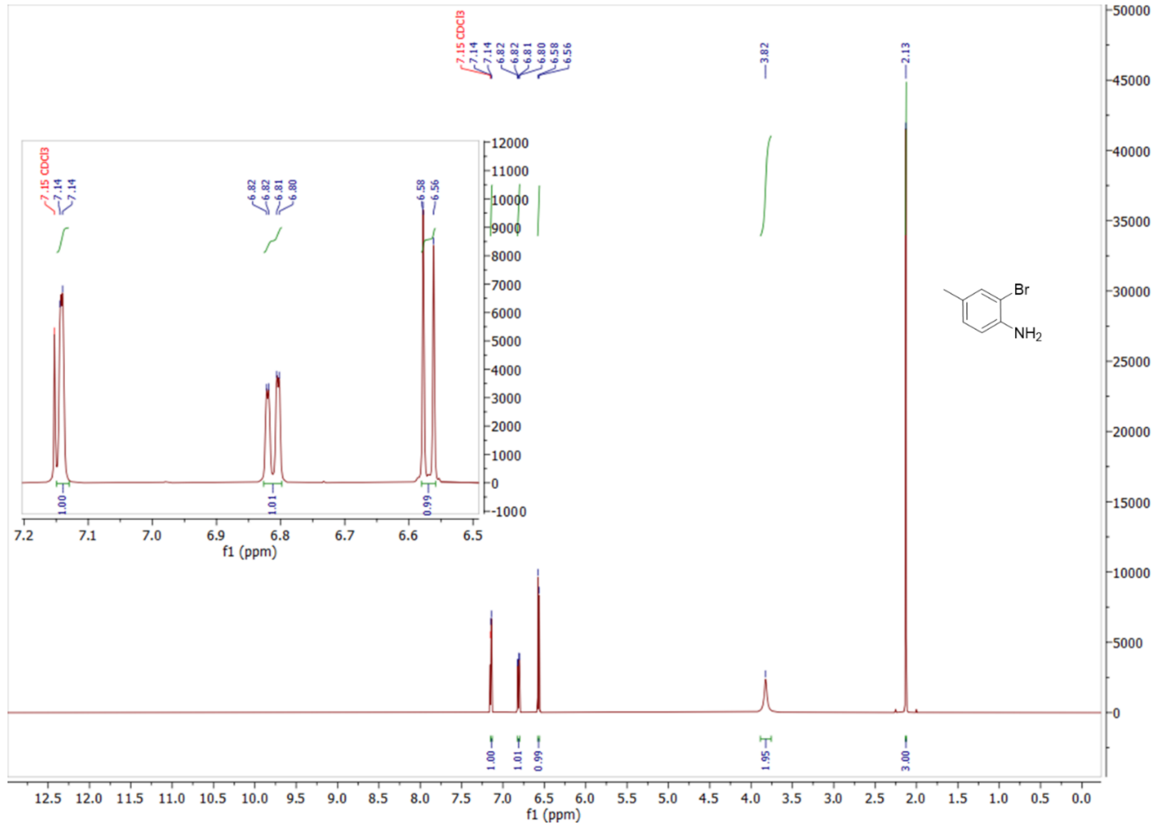

Figure S24: ^{1}H NMR (500 MHz, $CDCl_3$) of compound **6l**.

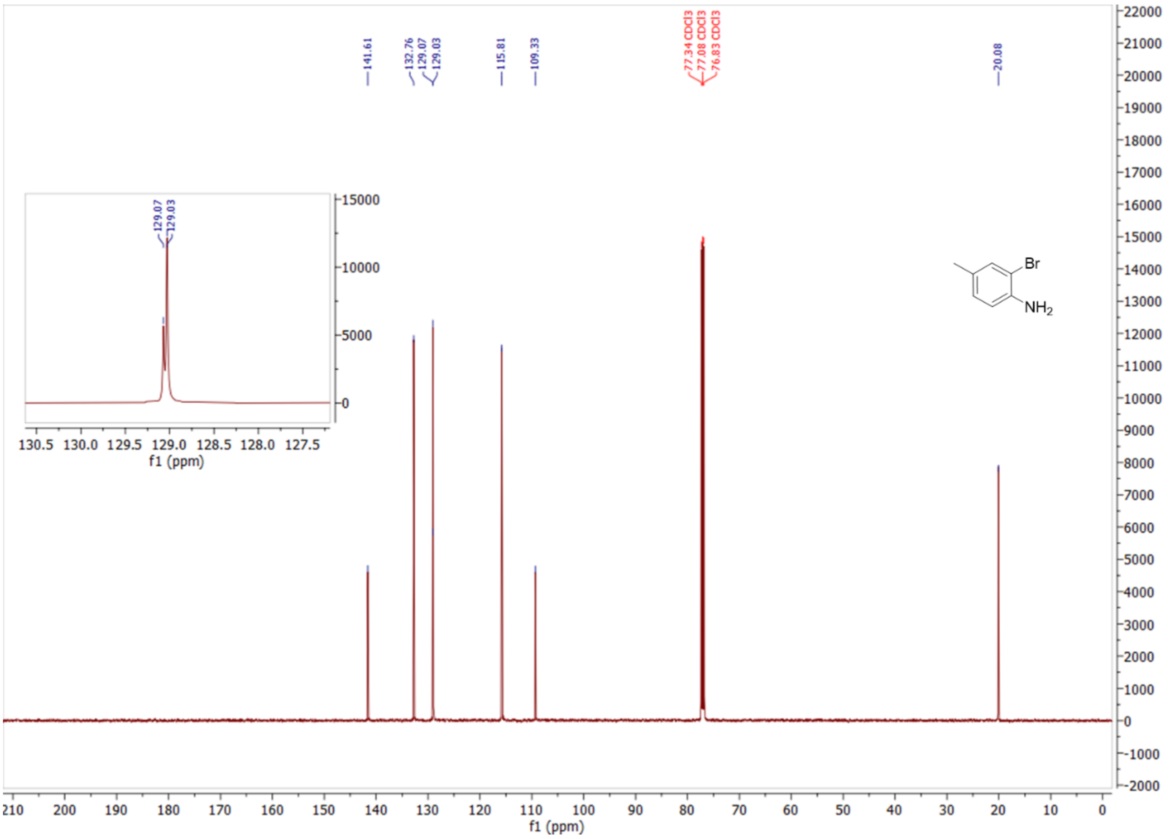

Figure S25: $^{13}C\{1H\}$ NMR (125 MHz, $CDCl_3$) of compound **6l**.

2-Bromo-4-(trifluoromethyl)aniline (6m)

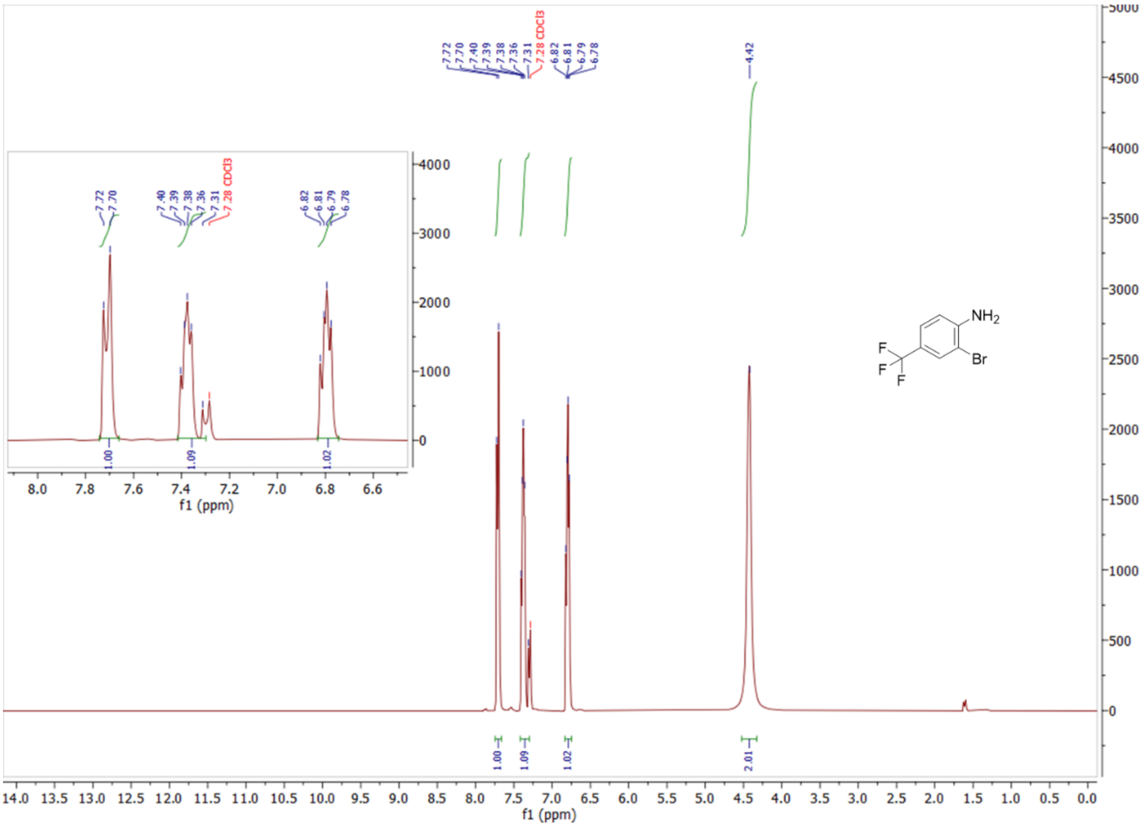

Figure S26: ^{1}H NMR (500 MHz, $CDCl_3$) of compound **6m**.

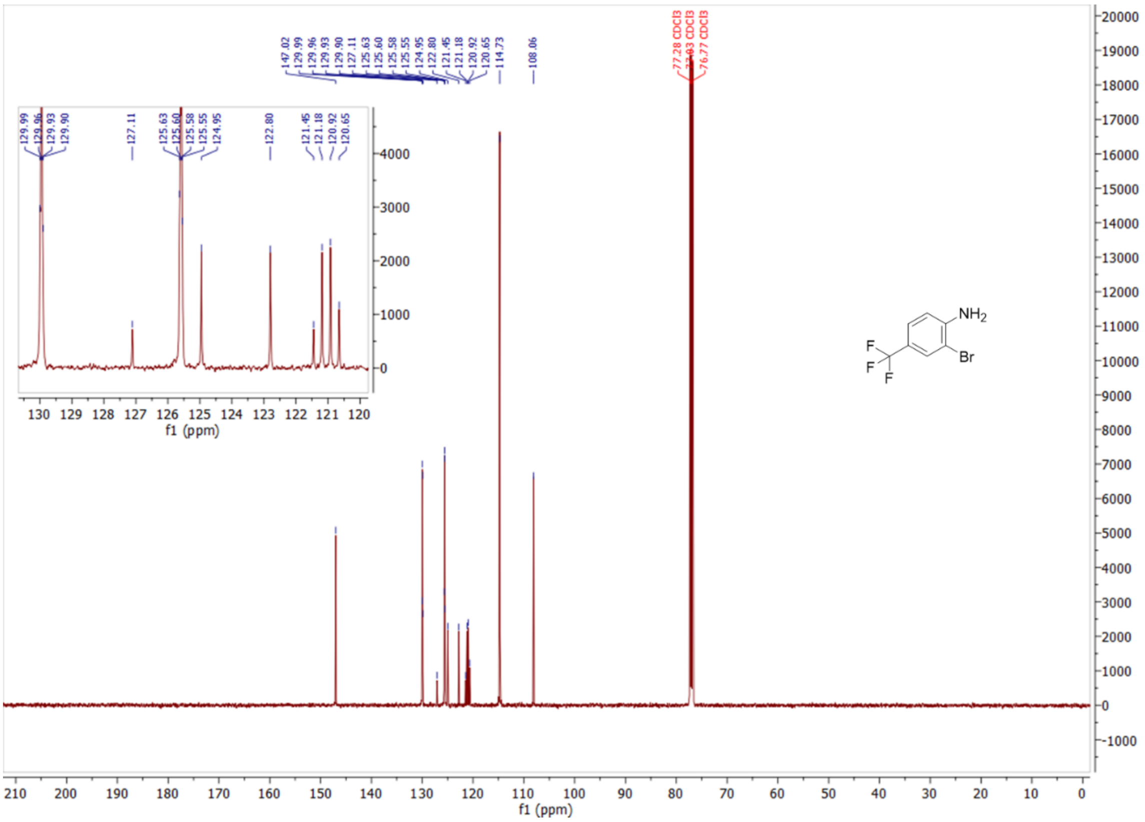

Figure S27: $^{13}C\{1H\}$ NMR (125 MHz, $CDCl_3$) of compound **6m**.

4-Bromo-1*H*-pyrazole (6n)

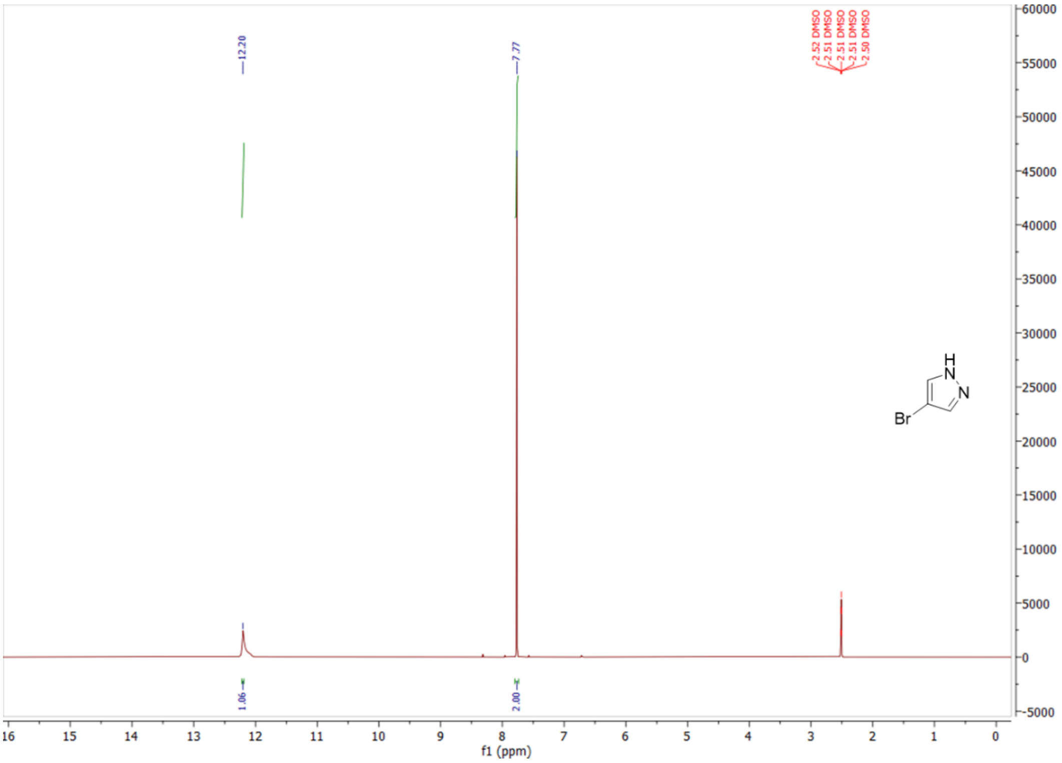

Figure S28: ^{1}H NMR (500 MHz, DMSO-d_6) of compound **6n**.

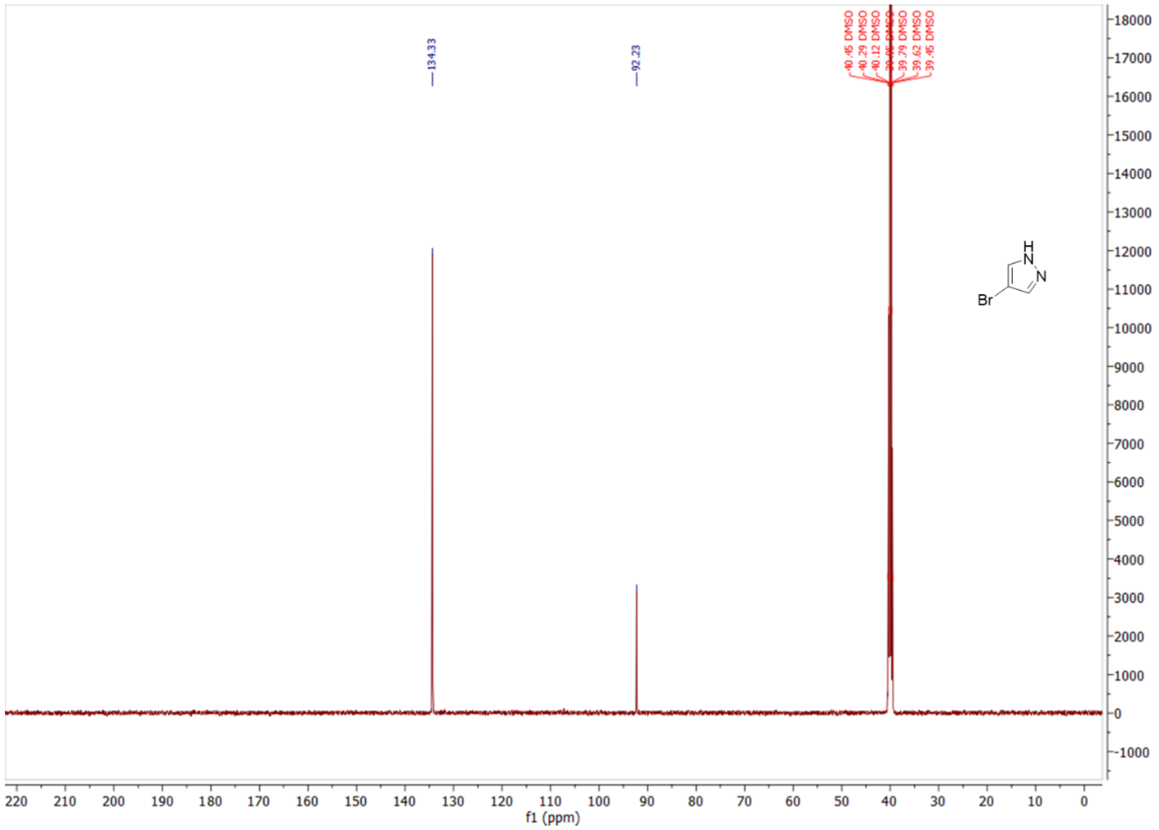

Figure S29: $^{13}C\{1H\}$ NMR (125 MHz, DMSO-d_6) of compound **6n**.

4-Bromo-3,5-dimethyl-1*H*-pyrazole (6o)

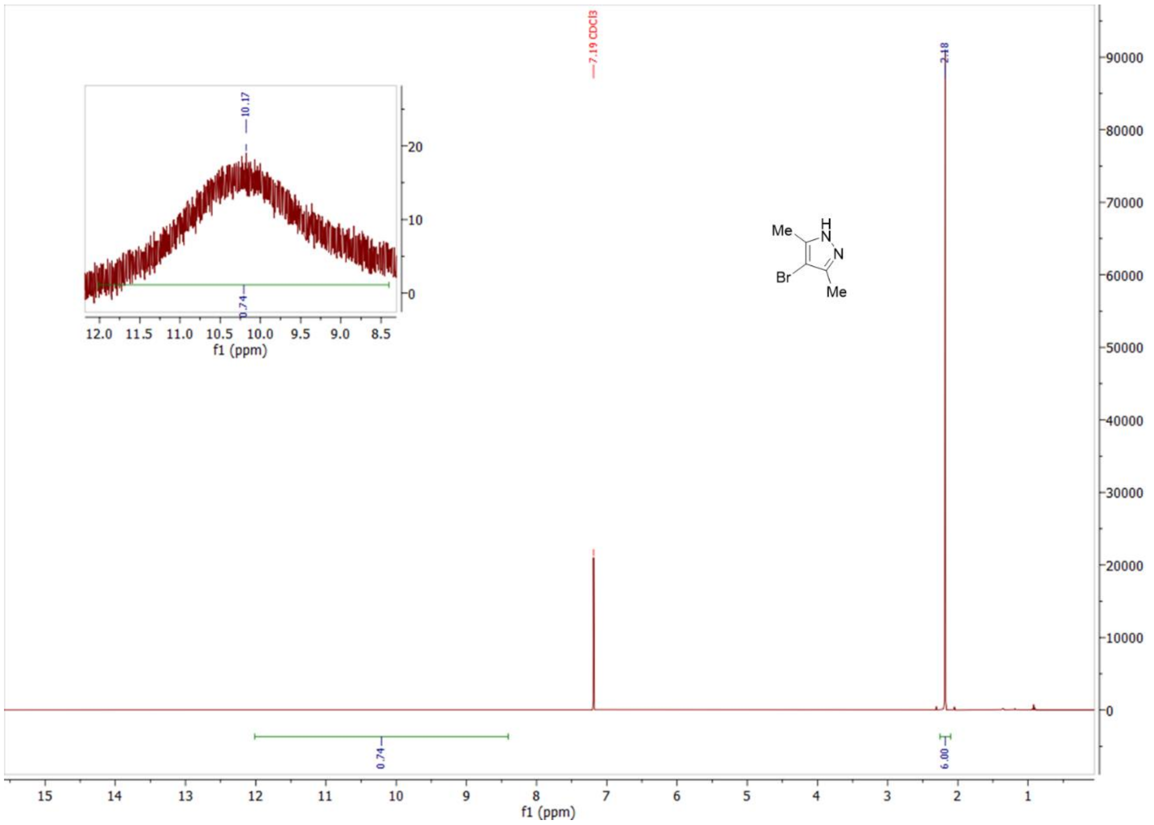

Figure S30: ^{1}H NMR (500 MHz, $CDCl_3$) of compound **6o**.

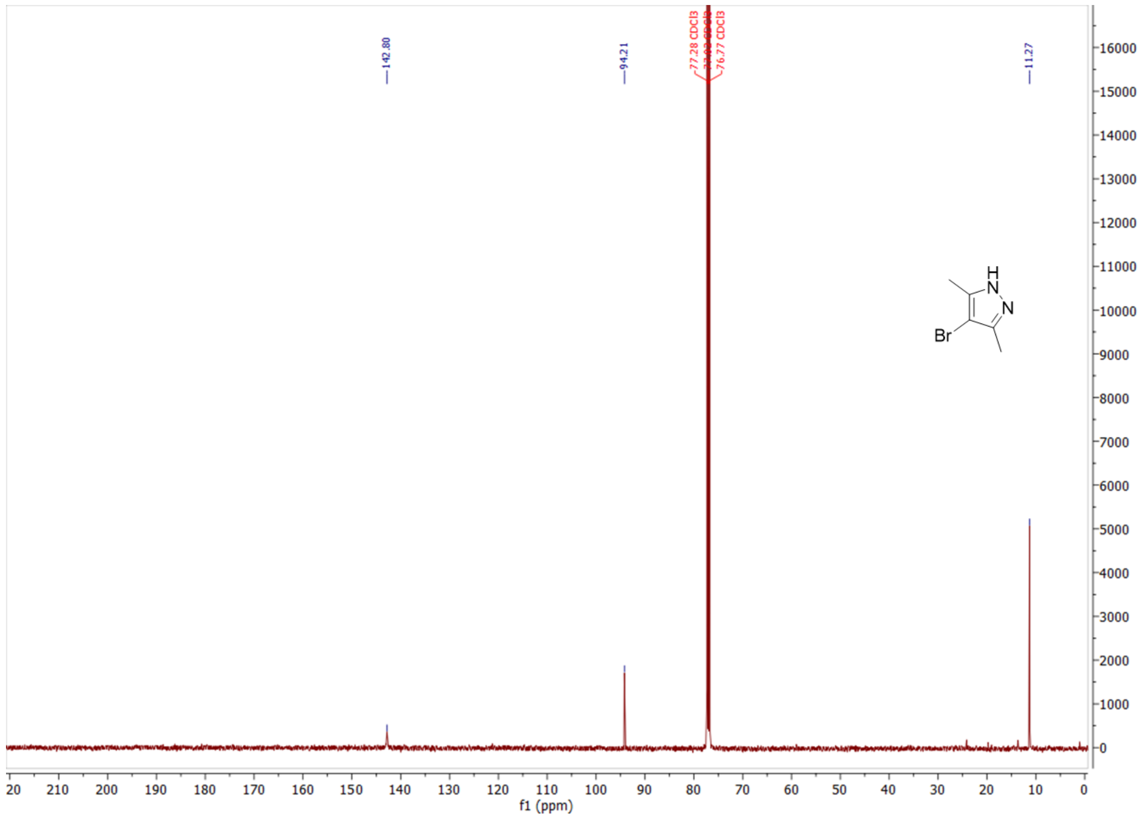

Figure S31: $^{13}C\{1H\}$ NMR (125 MHz, $CDCl_3$) of compound **6o**.

1,3-Dibromoazulene (6p)

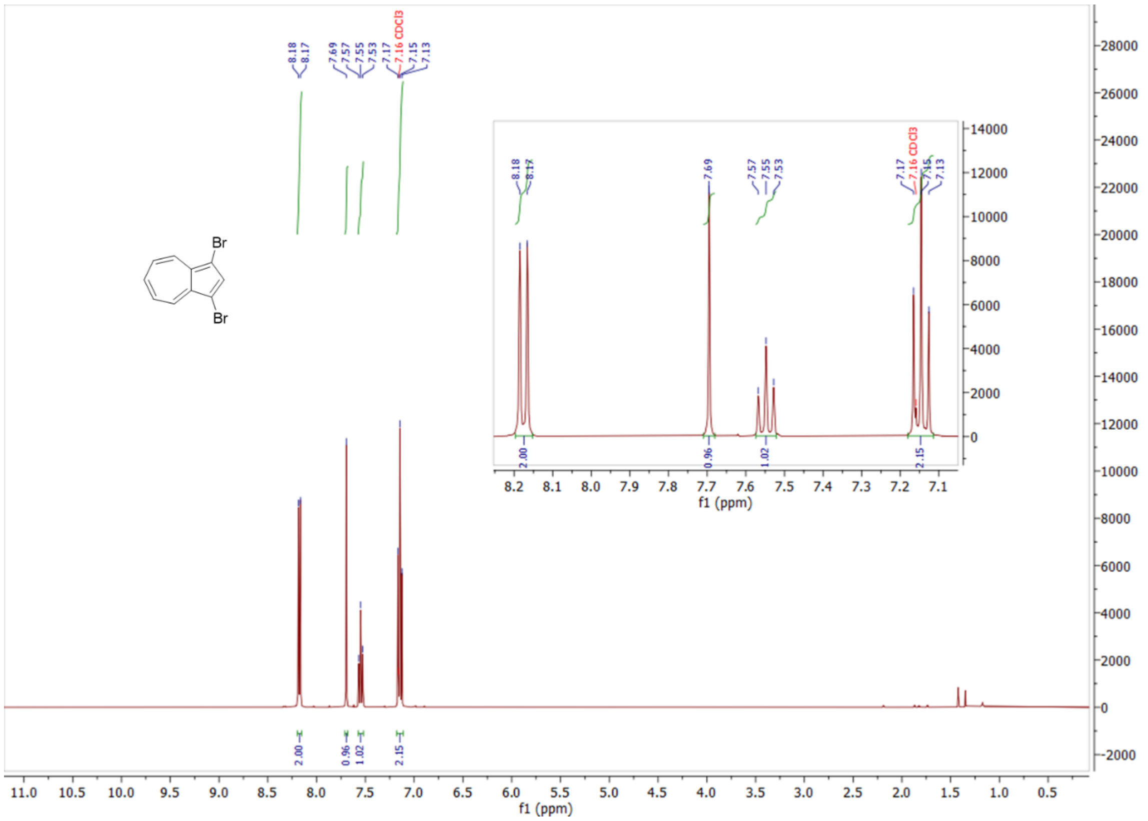

Figure S32: ^{1}H NMR (500 MHz, DMSO-d_6) of compound **6p**.

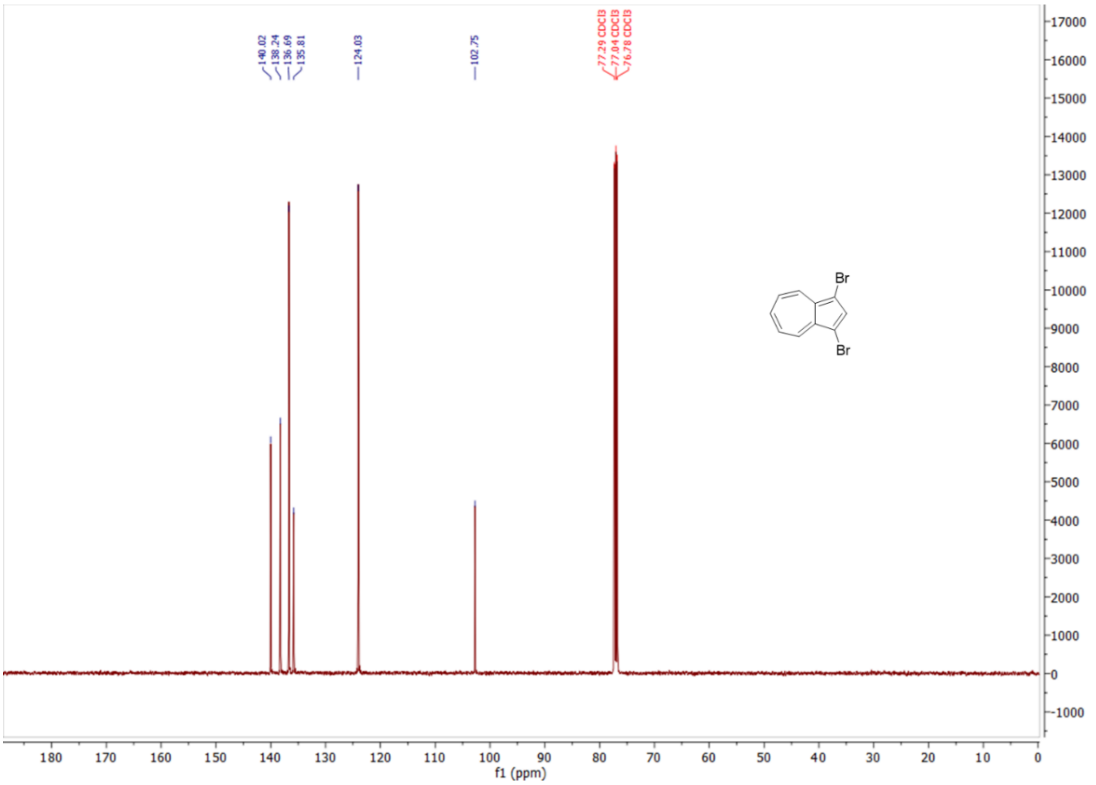

Figure S33: ^{13}C{1H} NMR (125 MHz, DMSO-d$_6$) of compound **6p.**

5,7-Dibromoquinolin-8-ol (6q)

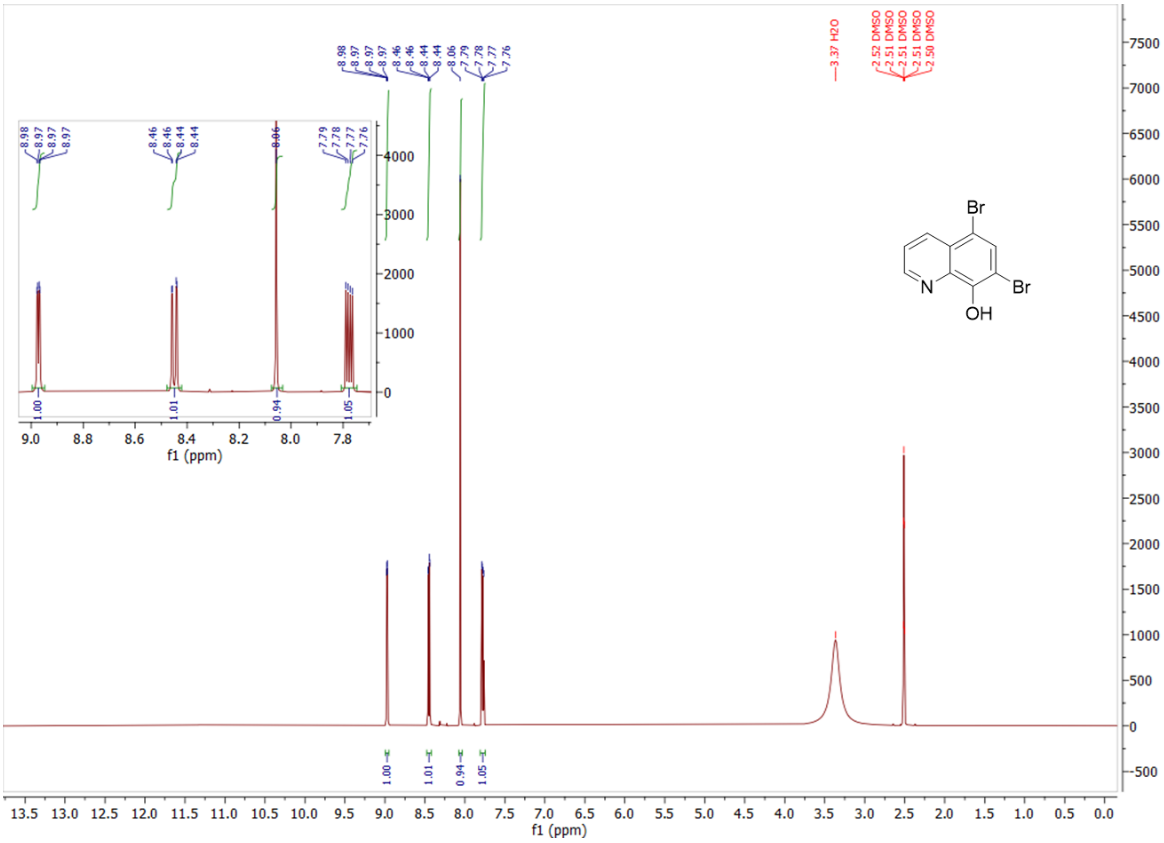

Figure S34: ^{1}H NMR (500 MHz, DMSO-d$_6$) of compound **6q**.

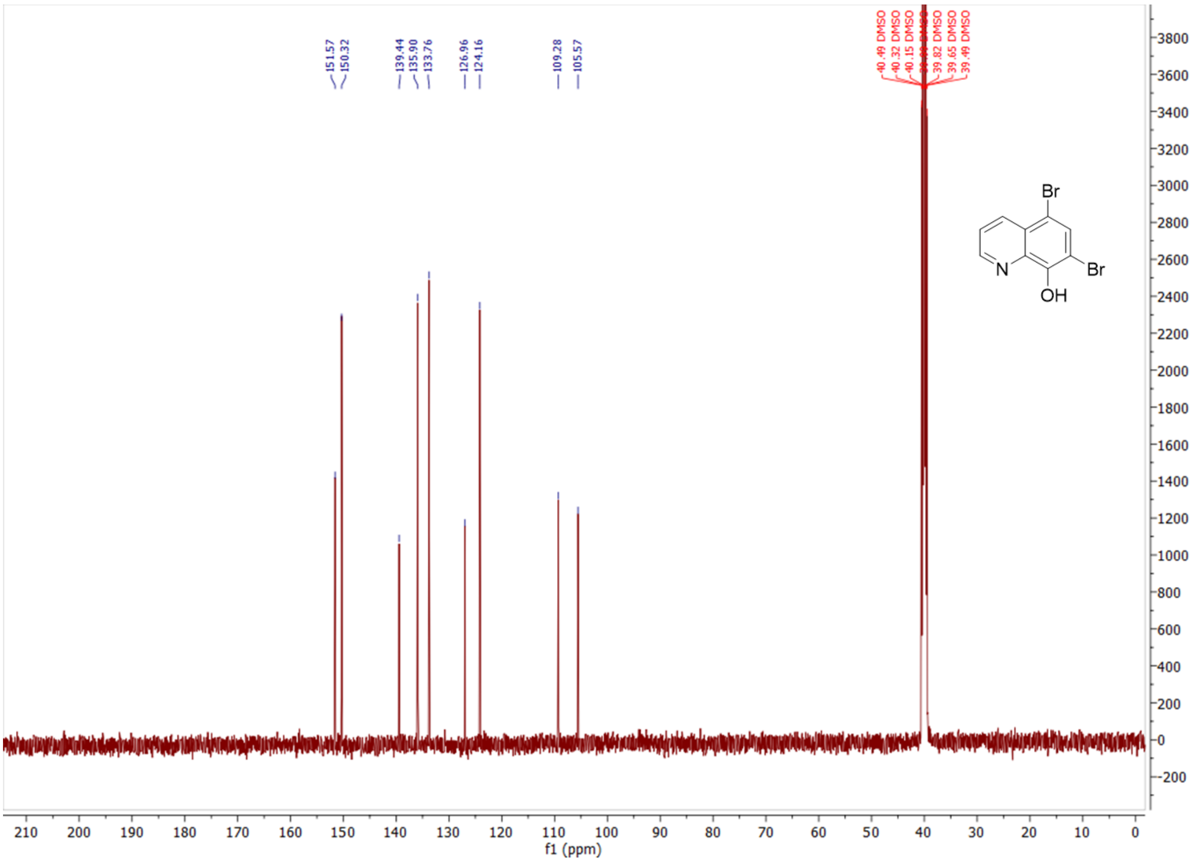

Figure S35: $^{13}C\{1H\}$ NMR (125 MHz, DMSO-d_6) of compound **6q.**

References

1) Tang, Y.; Yu, B. A mild heteroatom(O-, N-, and S-)methylation protocol using trimethyl phosphate (TMP)-$Ca(OH)_2$ combination. *Synthesis* **2022**, *54*, 2373-2390.
2) He, X.; Wang, X.; Tse, Y. L.; Ke, Z.; Yeung, Y. Y. Applications of selenonium cations as Lewis acids in organocatalytic reactions. *Angew. Chem. Int. Ed*. **2018**, *57*, 12869-12873.
3) Gavinolla, V.; Thangalipalli, S.; Bandalla, S. G.; Panduga, R.; Neella, C. K. A thermo-regulated highly regioselective mono and dihalogenations of phenols and anilines in water employing new Lewis base adducts (LBAs)$[DBUBr]^+$ Br^- and $[DBUI]^+$ I^- as green reagents: A simple approach. *New. J. Chem.* **2023**, *47*, 20777-20784.
4) Gao, X.; Geng, Y.; Han, S.; Liang, A.; Li, J.; Zou, D.; Wu, Y.; Wu, Y. Nickel-catalyzed direct C-H trifluoromethylation of free anilines with Togni's reagent. *Org. Lett.* **2018**, *20*, 3732-3735.
5) Yadav, I.; Prakash, V.; Maurya, M. R.; Sankar, M. Oxido-molybdenum(V) corroles as robust catalysts for oxidative bromination and selective epoxidation reactions in aqueous media under mild conditions. *Synthesis* **2023**, *62*, 5292-5301.
6) Mondal, H.; Patra, S.; Saha, S.; Nayak, T.; Sengupta, U.; Sudan Maji, M. Late-stage halogenation of peptides, drugs and (hetero)aromatic compounds with a nucleophilic hydrazide catalyst. *Angew. Chem. Int. Ed.* **2023**, *62*, e202312597.
7) Bhadra, S.; Dzik, W. I.; Gooßen, L. J. Synthesis of aryl ethers from benzoates through carboxylate-directed C-H-activating alkoxylation with concomitant protodecarboxylation. *Angew. Chem. Int. Ed.* **2013**, *10*, 2959-2962.
8) Howard, J. R.; Bhakare, A.; Akhtar, Z.; Wolf, C.; Anslyn, E. V. Data-driven prediction of circular dichroism-based calibration curves for the rapid screening of chiral primary amine enantiomeric excess values. *J. Am. Chem. Soc.* **2022**, *144*, 17269-17276.
9) Yadav, I.; Prakash, V.; Maurya, M. R.; Sankar, M. Oxido-molybdenum(V) corroles as robust catalysts for oxidative bromination and selective epoxidation reactions in aqueous media under mild conditions. *Inorg. Chem.* **2023**, *62*, 5292-5301.

10) Shukla, G.; Singh, M.; Yadav, A. K.; Singh, M. S. Aromatic C(sp^2)-H functionalization by consecutive paired electrolysis: Dibromination of aryl amines with dibromoethane at room temperature. *Chem. Eur. J.* **2023**, *30*, e202303179.
11) Xu, X. J.; Amuti, A.; Wusiman, A. Catalysis, Catalyst and additive-free direct amidation/halogenation of tertiary arylamines with *N*-haloimide/amides. *Org. Biomol. Chem.* **2020**, *362*, 5002-5008.
12) He, C.; Ma, F.; Zhang, W.; Tong, R. Reinvestigating $FeBr_3$-catalyzed alcohol oxidation with H_2O_2: Is a high-valent iron species (HIS) or a reactive brominating species (RBS) responsible for alcohol oxidation? *Org. Lett.* **2022**, *24*, 3499-3503.
13) Murakami, M.; Maeda, K.; Maeda, H.; Segi, M.; Furuyama, T. Synthesis of V-shaped fused phthalonitriles and control of their molecular orientation. *Tetrahedron Lett*. **2022**, *95*, 153750.
14) Bondarenko, O. B.; Karetnikov, G. L.; Komarov, A. I.; Pavlov, A. I.; Nikolaeva, S. N. $R_4NHal/NOHSO_4$: A usable system for halogenation of isoxazoles, pyrazoles, and beyond. *J. Org. Chem.* **2020**, *86*, 322-332.
15) Olsen, K. L.; Jensen, M. R.; MacKay, J. A. A mild halogenation of pyrazoles using sodium halide salts and oxone. *Tetrahedron Lett*. **2017**, *58*, 4111-4114.
16) Dubovik, J.; Bredihhin, A. A convenient synthesis of functionalized azulenes via Negishi cross-coupling. *Synthesis* **2015**, *47*, 538-548.
